# Gasdermin D Deficiency in Vascular Smooth Muscle Cells Ameliorates Abdominal Aortic Aneurysm Through Reducing Putrescine Synthesis

**DOI:** 10.1002/advs.202204038

**Published:** 2022-12-25

**Authors:** Jianing Gao, Yanghui Chen, Huiqing Wang, Xin Li, Ke Li, Yangkai Xu, Xianwei Xie, Yansong Guo, Nana Yang, Xinhua Zhang, Dong Ma, Hong S. Lu, Ying H. Shen, Yong Liu, Jifeng Zhang, Y. Eugene Chen, Alan Daugherty, Dao Wen Wang, Lemin Zheng

**Affiliations:** ^1^ The Institute of Cardiovascular Sciences and Institute of Systems Biomedicine School of Basic Medical Sciences Key Laboratory of Molecular Cardiovascular Science of Ministry of Education NHC Key Laboratory of Cardiovascular Molecular Biology and Regulatory Peptides Beijing Key Laboratory of Cardiovascular Receptors Research Health Science Center Peking University Beijing 100191 P. R. China; ^2^ Division of Cardiology Department of Internal Medicine and Hubei Key Laboratory of Genetics and Molecular Mechanism of Cardiologic Disorders Tongji Hospital Tongji Medical College Huazhong University of Science and Technology Jiefang Avenue NO.1095, Qiaokou District Wuhan 430000 P. R. China; ^3^ Beijing Tiantan Hospital China National Clinical Research Center for Neurological Diseases Advanced Innovation Center for Human Brain Protection Beijing Institute of Brain Disorders The Capital Medical University Beijing 100050 P. R. China; ^4^ Department of Cardiology Shengli Clinical Medical College of Fujian Medical University Fujian Provincial Hospital Fuzhou 350001 P. R. China; ^5^ Department of Cardiology Shengli Clinical Medical College of Fujian Medical University Fujian Provincial Hospital Fujian Provincial Key Laboratory of Cardiovascular Disease Fujian Provincial Center for Geriatrics Fujian Clinical Medical Research Center for Cardiovascular Diseases Fujian Heart Failure Center Alliance Fuzhou 350001 P. R. China; ^6^ Weifang Key Laboratory of Animal Model Research on Cardiovascular and Cerebrovascular Diseases Weifang Medical University Weifang 261053 P. R. China; ^7^ Department of Biochemistry and Molecular Biology The Key Laboratory of Neural and Vascular Biology Ministry of Education Hebei Medical University Zhongshan East Road No. 361 Shijiazhuang 050017 P. R. China; ^8^ Department of Biochemistry and Molecular Biology The Key Laboratory of Neural and Vascular Biology China Administration of Education Hebei Medical University Hebei 050017 P. R. China; ^9^ Department of Physiology Saha Cardiovascular Research Center University of Kentucky South Limestone Lexington KY 40536‐0298 USA; ^10^ Division of Cardiothoracic Surgery Michael E. DeBakey Department of Surgery Baylor College of Medicine Department of Cardiovascular Surgery Texas Heart Institute Houston TX 77030 USA; ^11^ Hubei Key Laboratory of Cell Homeostasis College of Life Sciences Institute for Advanced Studies Wuhan University Wuhan 430072 P. R. China; ^12^ Department of Internal Medicine University of Michigan Medical Center Ann Arbor MI 48109 USA; ^13^ Hangzhou Qianjiang Distinguished Expert Hangzhou Institute of Advanced Technology Hangzhou 310026 P. R. China

**Keywords:** abdominal aortic aneurysm, gasdermin D, putrescine, smooth muscle cells

## Abstract

Abdominal aortic aneurysm (AAA) is a common vascular disease associated with significant phenotypic alterations in vascular smooth muscle cells (VSMCs). Gasdermin D (GSDMD) is a pore‐forming effector of pyroptosis. In this study, the role of VSMC‐specific GSDMD in the phenotypic alteration of VSMCs and AAA formation is determined. Single‐cell transcriptome analyses reveal *Gsdmd* upregulation in aortic VSMCs in angiotensin (Ang) II‐induced AAA. VSMC‐specific *Gsdmd* deletion ameliorates Ang II‐induced AAA in apolipoprotein E (ApoE)^−/−^ mice. Using untargeted metabolomic analysis, it is found that putrescine is significantly reduced in the plasma and aortic tissues of VSMC‐specific GSDMD deficient mice. High putrescine levels trigger a pro‐inflammatory phenotype in VSMCs and increase susceptibility to Ang II‐induced AAA formation in mice. In a population‐based study, a high level of putrescine in plasma is associated with the risk of AAA (*p* < 2.2 × 10^−16^), consistent with the animal data. Mechanistically, GSDMD enhances endoplasmic reticulum stress‐C/EBP homologous protein (CHOP) signaling, which in turn promotes the expression of ornithine decarboxylase 1 (ODC1), the enzyme responsible for increased putrescine levels. Treatment with the ODC1 inhibitor, difluoromethylornithine, reduces AAA formation in Ang II‐infused ApoE^−/−^ mice. The findings suggest that putrescine is a potential biomarker and target for AAA treatment.

## Graphical abstract

This work reveals that GSDMD in VSMCs contributes to abdominal aortic aneurysm (AAA) by upregulating plasma putrescine concentrations. GSDMD stimulates ER stress and triggers the putrescine synthesis in VSMCs. High putrescine switches VSMC phenotype to synthetic phenotype that contributes to AAA. The manuscript suggests that putrescine is a potential biomarker and target for AAA treatment.

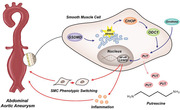

## 1 Introduction

Abdominal aortic aneurysm (AAA) is a common vascular disease that is associated with a high risk of death. Rupture of an aortic aneurysm is often lethal, with mortality rates of 85–90%.^[^ ^1^ ^]^ Male gender, older age, smoking, atherosclerosis, and family history of the disease are the principal risk factors for AAA. AAA is accompanied by chronic inflammation, vascular smooth muscle remodeling and apoptosis, and extravascular matrix (ECM) degradation.^[^ ^2^ ^]^ Currently, there is no approved medical therapy for AAA, making prevention and early diagnosis particularly critical.^[^ ^3^ ^]^ Phenotype switching and apoptosis of vascular smooth muscle cells (VSMCs) are important pathological hallmarks.^[^ ^4^ ^]^ Accumulating evidence suggests that active metabolites can alter cell physiology;^[^ ^5^ ^]^ however, the VSMC metabolites involved in AAA have not yet been fully defined.

Gasdermin D (GSDMD) is a pore‐forming effector of pyroptosis.^[^ ^6^ ^]^ Inflammasome complexes caspase‐11 and caspase‐8 lead to GSDMD cleavage; the GSDMD N‐terminal domain N‐GSDMD subsequently oligomerizes and forms pores in the plasma membrane of macrophages.^[^ ^7^ ^]^ Pharmacological inhibition of GSDMD is efficacious in mice with sepsis.^[^ ^8^ ^]^ It has also been reported that loss of GSDMD results in improved ventricular remodeling and reduced heart failure after acute myocardial infarction.^[^ ^9^ ^]^ GSDMD also mediates cardiomyocyte pyroptosis during myocardial ischemia‐reperfusion injury.^[^ ^10^ ^]^ Therefore, GSDMD plays an important role in the development of cardiovascular disease. However, the effects of GSDMD on VSMCs with respect to AAA have not yet been studied.

Here, we report that GSDMD promotes AAA by inducing endoplasmic reticulum (ER) stress and upregulating C/EBP homologous protein (CHOP) to increase putrescine synthesis. Plasma putrescine concentrations positively correlated with human AAA, whereas treatment with difluoromethylornithine (DFMO), an inhibitor of putrescine, partially prevented AAA development.

## 2 Results

### 2.1 VSMC‐Specific GSDMD Deficiency Reduced AAA

**Figure 1:**
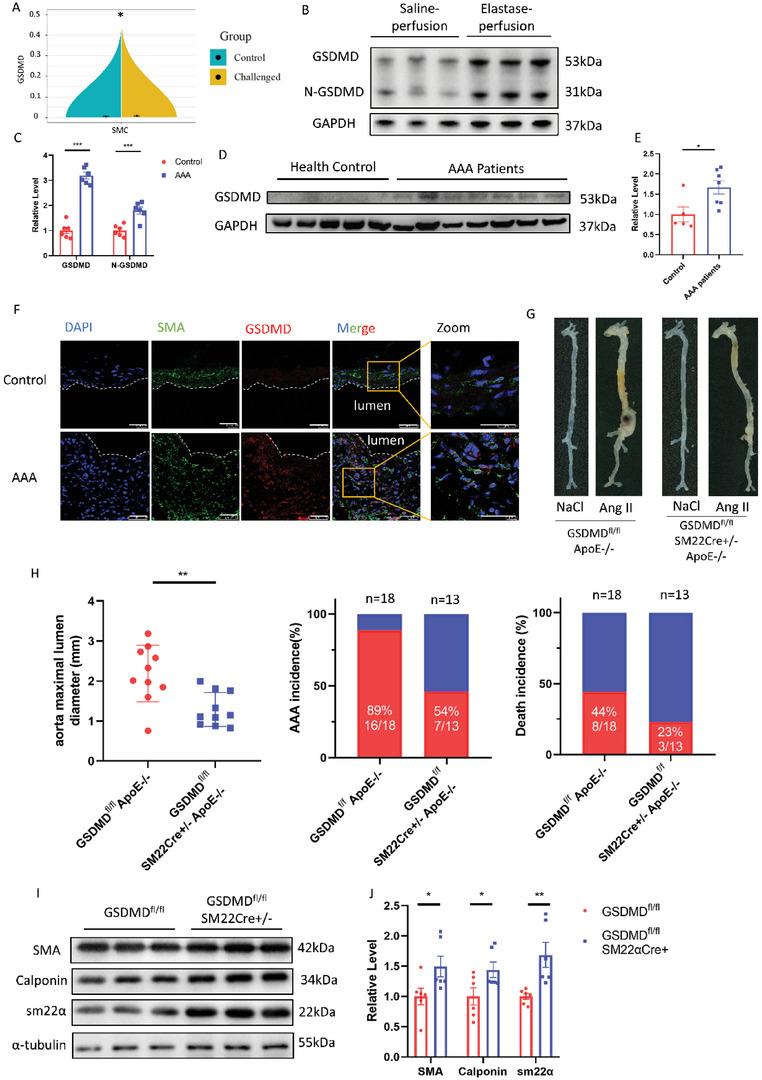
VSMC‐specific GSDMD deficiency reduced abdominal aortic aneurysm. A) RNA‐sequencing (RNA‐seq) analysis of GSDMD was performed in ascending aortas from wild‐type (WT) mice that were control or fed a high‐fat diet for 5 weeks and Ang II (2000 ng/min/kg) infusion during the last week. B,C) Representative western blotting (B) and quantification (C) of GSDMD and N‐GSDMD from aortas of C57BL/6J mice with 14 days postperfusion of elastase or saline. *n* = 6 per group. Student's *t* test, **p  <  0.01. D,E) Representative western blotting (D) and quantification (E) of GSDMD from aortas of human normal aortas and AAA patients. *n* = 5 for healthy group and *n* = 7 for AAA patients’ group. Student's *t‐*test, *p  <  0.05. F) Representative immunofluorescence staining of GSDMD (red) and SMA (green) in control and AAA mice aortas, scale bar = 50 µm. G,H) Twenty‐eight‐day infusion of Ang II (1000 ng/min/kg) or saline in 4‐month‐old GSDMD^fl/fl^SM22Cre^+/−^ApoE^−/−^ and GSDMD^fl/fl^ApoE^−/−^ male mice. Representative morphology of the aortas (G). The quantification of maximal suprarenal aortic diameters measured ex vivo, the incidence of AAA, and the death rate of mice. The red part in histogram is the incidence and death rate of AAA model mice. The blue part in histogram is the non‐disease and survival rate of AAA model mice (H). *n* = 10 per group. Student's *t* test, **p  <  0.01. I,J) Representative western blotting (I) and quantification (J) of aortas of GSDMD^fl/fl^SM22Cre^+/−^ and GSDMD^fl/fl^ mice with 14 days postperfusion of elastase or saline. *n* = 6 per group. Student's *t‐*test, *p  <  0.05, **p  <  0.01.

Through analysis of single‐cell transcriptome data from Luo et al.,^[^ ^11^ ^]^ we identified increased *Gsdmd* expression in the VSMCs of aortic aneurysm and dissection (AAD) tissues from mice treated with a high‐fat diet and angiotensin (Ang) II, compared to those from aortic tissues from control mice (**Figure** 1A). Western blot analysis found increased GSDMD and N‐GSDMD level in the AAA tissue of male wild‐type mice perfused with elastase at 12 weeks of age compared to those in saline‐perfused mice (Figure 1B,C). GSDMD expression was also increased in the aortas of AAA patients compared to those in healthy controls (Figure 1D,E; Figure S1A and Table S1, Supporting Information). Immunofluorescence revealed increased GSDMD expression in VSMCs of mouse AAA samples compared to that in saline‐treated, respectively (Figure 1F). To determine whether ablation of VSMC‐derived GSDMD ameliorated AAA, we generated VSMC‐restricted GSDMD‐deficient mice and crossed VSMC‐restricted GSDMD‐deficient mice with the apolipoprotein E (ApoE) knockout mice; that is, GSDMD^fl/fl^SM22Cre^+/−^, GSDMD^fl/fl^, GSDMD^fl/fl^SM22Cre^+/−^ApoE^−/−^, and GSDMD^fl/fl^ApoE^−/−^ mice (Figure S1B, Supporting Information). Four‐month‐old male GSDMD^fl/fl^SM22Cre^+/−^ApoE^−/−^ and GSDMD^fl/fl^ApoE^−/−^mice were infused with Ang II (1000 ng/kg/min) for 28 days, and the models were verified by their elevated blood pressure results (Figure S1C, Supporting Information). Our results showed that the maximal ex vivo diameters of suprarenal aortas were smaller and AAA incidence and death rates were lower in GSDMD^fl/fl^SM22Cre^+/−^ApoE^−/−^ mice compared to GSDMD^fl/fl^ApoE^−/−^ mice (Figure 1G,H). Elastin fragmentation was reduced in the aortic media from GSDMD^fl/fl^SM22Cre^+/−^ApoE^−/−^ mice compared to that in their GSDMD^fl/fl^ApoE^−/−^ littermates, as determined by Elastin Van Gieson (EVG) staining (Figure S1D,E, Supporting Information). In the elastase induction model of AAA, the aortas were also significantly less expanded in GSDMD^fl/fl^SM22Cre^+/−^mice than in the GSDMD^fl/fl^ mice (Figure S1F–I, Supporting Information). VSMC‐specific GSDMD deficiency resulted in higher levels of expression of *α*‐smooth muscle actin, smooth muscle 22, and calponin (Figure 1I,J). These three contractile proteins were also upregulated in human aortic smooth muscle cells (HASMCs) with small interfering RNA (siRNA)‐induced GSDMD knockdown (Figure S1J–M, Supporting Information). Collectively, these results suggest that VSMC‐specific GSDMD deficiency reduces the incidence of AAA in mice and preserves the contractile phenotype of VSMCs.

### 2.2 Putrescine Was Upregulated in AAA

**Figure 2:**
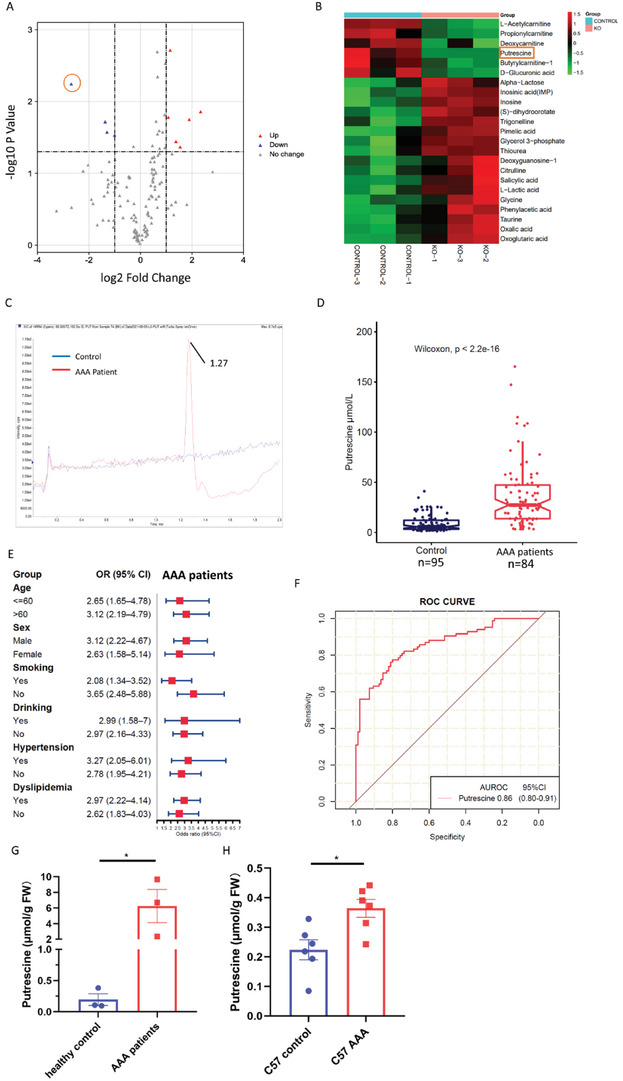
Putrescine upregulated in AAA patients’ plasma. A,B) Volcano plot (A) and heatmap (B) illustrating differential metabolites between the aortas of GSDMD^fl/fl^SM22Cre^+/−^ and GSDMD^fl/fl^ mice. The orange box shows the putrescine. *n* = 3. C) Representative extracted ion chromatogram for putrescine of healthy control and AAA patients. D) Putrescine concentrations of human plasma from 95 healthy controls and 84 AAA patients. Wilcoxon test. E) Forest plot showing the per‐SD OR (95% CI) for plasma putrescine concentrations associated with AAA in subgroups. Logistic regression. F) ROC curves: AAA versus healthy controls for putrescine. G) Putrescine concentrations of human normal aortas and aneurysmal aortas patients. *n* = 3 per group. FW, fresh weight. Student's *t‐*test, *p  <  0.05. H) Putrescine of C57BL/6J mice aortas with 14 days postperfusion of elastase or saline. *n* = 6 per group. FW, fresh weight. Student's *t‐*test, *p <  0.05.

To explore which metabolites could affect VSMC homeostasis, untargeted metabolomic studies of the abdominal aorta were performed in mice perfused with elastase for 14 days. The levels of the metabolite putrescine showed the greatest reduction in the aortas of the GSDMD^fl/fl^SM22Cre^+/−^group compared to those of the GSDMD^fl/fl^ group (**Figure** **2**A,B). Putrescine is a polyamine that is synthesized from ornithine via decarboxylation of the amino acid.^[^ ^12^ ^]^ High concentrations of putrescine have been detected in cancer and inflammatory bowel diseases.^[^ ^13^, ^14^ ^]^ Metabolomic phenotyping of experimental animal models of AAA has shown that putrescine is upregulated in the blood and aortas of Ang II‐induced mice;^[^ ^15^ ^]^ however, the role of putrescine in aortic aneurysms remains unclear.

To identify whether putrescine is involved in the etiology of AAA in humans, we performed a case‐control study matched for age, sex, blood pressure, smoking status, alcohol use, and hypertension status in 84 patients with AAA and 95 healthy controls (Table S2, Supporting Information). Putrescine concentrations were significantly elevated in the plasma of AAA patients (median: 27.36 µmol L^−1^, interquartile range [IQR]: 13.80–47.32) compared with those in healthy controls (median: 5.69 µmol L^−1^, IQR: 3.35–12.18; Figure 2C,D, *P* < 2.22 × 10^−16^). Higher concentrations of plasma putrescine were associated with an increased risk of AAA across different strata subgroups defined by age, sex, smoking status, alcohol use, and history of hypertension or dyslipidemia (Figure 2E). In addition, the area under the receiver operator characteristic curve (AUROC) showed discriminating power with respect to predicting AAA risk (AUROC = 0.86, 95% confidence intervals [CI]: 0.80–0.91, sensitivity = 77.4%, specificity = 80%; Figure 2F). Putrescine concentrations were higher in the aortic tissues of AAA patients compared to that in healthy controls (Figure 2G). In addition, putrescine levels in the aortic tissues of C57BL/6J mice 14 days post‐perfusion with elastase were also higher than those in the tissues from the saline‐treated group (Figure 2H).

### 2.3 High Putrescine Levels Triggered the Development of AAA

**Figure 3:**
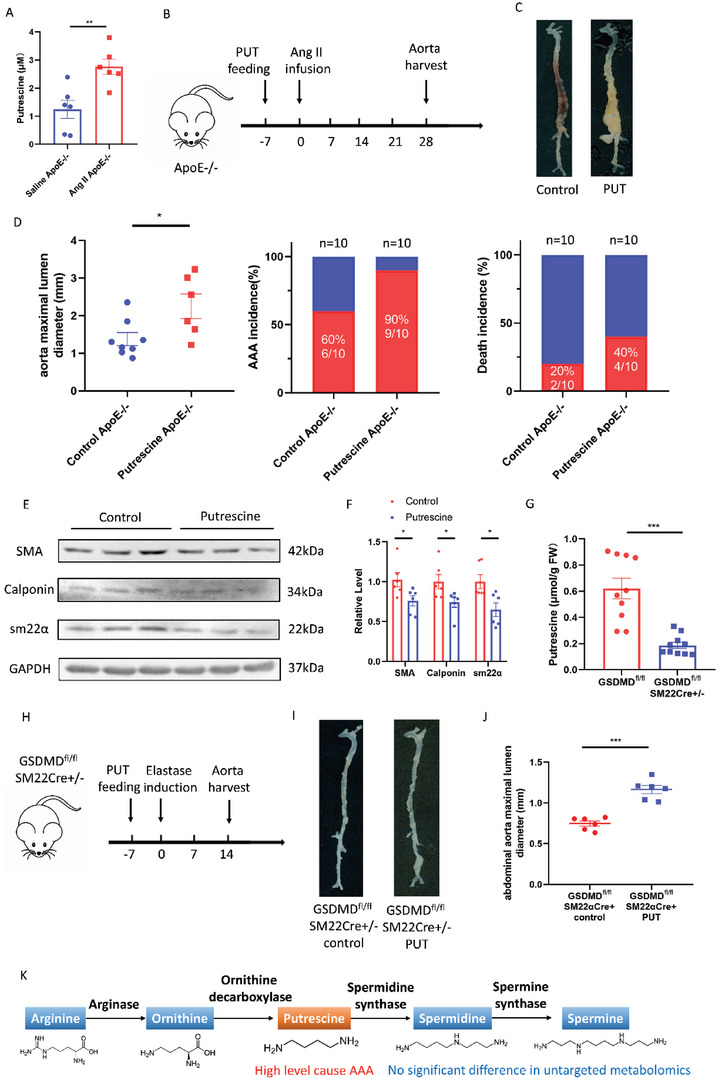
A high level of putrescine caused AAA via VSMCs phenotype switching. A–D) Twenty‐eight‐day infusion of Ang II or saline in 3‐month‐old ApoE^−/−^ male mice challenged with or without 1% putrescine in drinking water. Putrescine concentrations of ApoE^−/−^ mice plasma challenged with Ang II or saline. *n* = 6 per group. Student's *t*‐test, **p <  0.01 (A). The timeline of putrescine feeding and Ang II infusion (B). Representative morphology of the aortas (C). The quantification of maximal suprarenal aortic diameters measured ex vivo, the incidence of AAA, and the death rate of mice. The red part in a histogram is the incidence and death rate of AAA model mice. The blue part in a histogram is the non‐disease and survival rate of AAA model mice (D). *n* = 8 for water‐administered and *n* = 6 for putrescine‐administered. Student's *t*‐test, *p <  0.05. E,F) Representative western blotting (E) and quantification (F) from aortas of C57BL/6J mice challenged with or without 1% putrescine in drinking water with 14 days postperfusion of elastase. Student's *t‐*test, *p <  0.05. G) Putrescine concentrations of aortas of GSDMD^fl/fl^SM22Cre^+/−^ and GSDMD^fl/fl^ mice with 14 days postperfusion of elastase. FW, fresh weight. Student's *t‐*test, *n* = 10 per group, ***p <  0.001. H–J) Fourteen‐day postperfusion of elastase in 3‐month‐old GSDMD^fl/fl^SM22Cre^+/−^ male mice challenged with or without 1% putrescine in drinking water. *n* = 6 per group. The timeline of putrescine feeding and elastase induction (H). Representative morphology of the aortas (I). The quantification of maximal infrarenal aortic diameters measured ex vivo. Student's *t‐*test, ***p <  0.001 (J). K) Schematic diagram of the putrescine metabolic pathway.

To determine whether putrescine contributes to AAA, 3‐month‐old male ApoE^−/−^ mice were challenged with 1% putrescine in drinking water, followed 1 week later by Ang II infusion for 28 days. In addition to increased putrescine concentrations in the plasma, maximal suprarenal aortic diameters were larger in the putrescine‐administered mice infused with Ang II than in control mice administered pure drinking water prior to Ang II infusion. The death rate was also higher in the putrescine‐administered group compared to the water‐administered group (**Figure** **3**A–D). Elastin fragmentation was increased in the aortas of putrescine‐administered mice compared to water‐administered mice, as determined by EVG staining (Figure S2A,B, Supporting Information). The same results were observed in the C57BL/6J mice challenged with 1% putrescine in drinking water, followed 1 week later by elastase perfusion for 14 days (Figure S2C–G, Supporting Information). The expression levels of contractile proteins *α*‐smooth muscle actin, smooth muscle 22, and calponin were decreased in the aortas of putrescine‐administered mice and putrescine‐stimulated HASMCs, compared to the control groups (Figure 3E,F; Figure S2H,I, Supporting Information). In the elastase induction model, putrescine was downregulated in aorta tissues of GSDMD^fl/fl^SM22Cre^+/−^ mice compared to that in GSDMD^fl/fl^ mice (Figure 3G). A reduction in putrescine was also observed in HASMCs incubated with GSDMD siRNA compared to that in control cells (Figure S2J, Supporting Information).

Next, we investigated whether putrescine could reverse the protective effects of GSDMD deficiency in murine AAA. Higher maximal infrarenal aortic diameters were observed in GSDMD^fl/fl^SM22Cre^+/−^ mice perfused with elastase and administered 1% putrescine in drinking water compared to those perfused with elastase and administered water only (Figure 3H–J). Elastin fragmentation was also increased in the media of aortas of putrescine‐administered GSDMD^fl/fl^SM22Cre^+/−^ mice compared to mice of the same genotype administered water only, as determined by EVG staining (Figure S2K,L, Supporting Information). These data demonstrate that putrescine promotes the loss of the contractile phenotype of VSMCs and exacerbates AAA (Figure 3K).

### 2.4 Ornithine Decarboxylase 1 Promoted the Development of AAA

**Figure 4:**
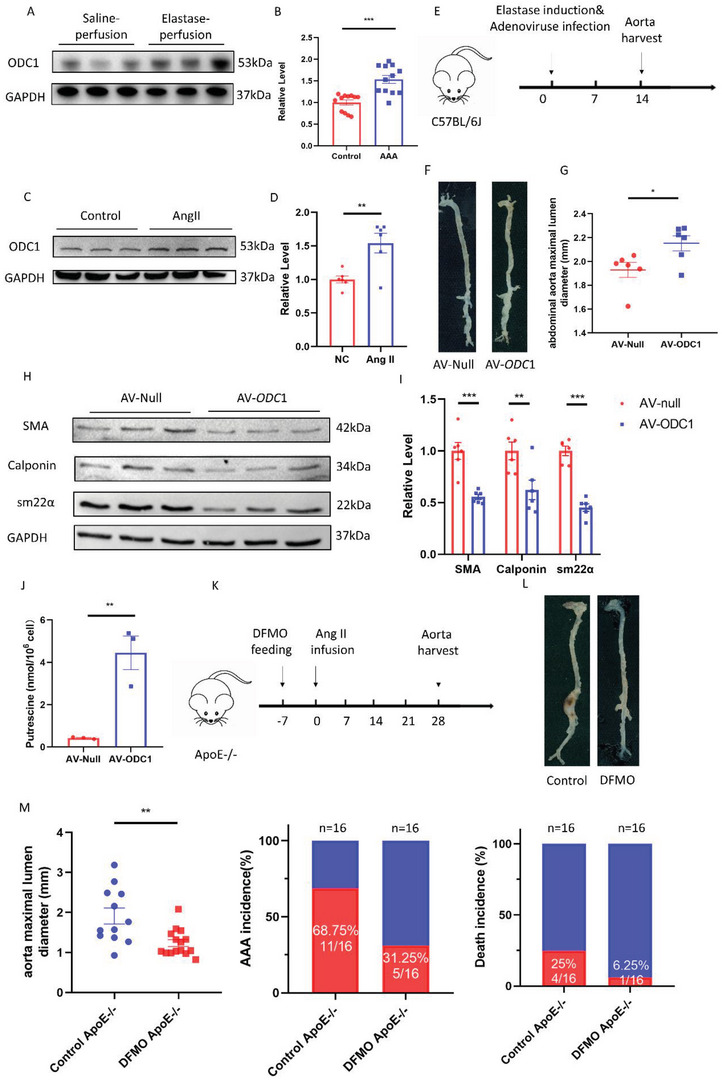
ODC1 promoted AAA development and inhibition of ODC1 by DFMO rescued AAA. A,B) Representative western blotting (A) and quantification (B) of ODC1 from aortas of C57BL/6J mice with 14 days postperfusion of elastase or saline. *n* = 12 per group. Student's *t‐*test, *p <  0.05. C,D) Representative western blotting (C) and quantification (D) of ODC1 from HASMCs incubated with or without 100 nm Ang II for 24 h. *n* = 6 per group. Student's *t‐*test, **p <  0.01. E–G) Fourteen‐day postperfusion of elastase in 3‐month‐old C57BL/6J male mice whose abdominal aortas periadventitially infected with Ad‐null and Ad‐*ODC1. n* = 6 per group. The timeline of adenovirus infection and elastase induction (E). Representative morphology of the aortas (F). The quantification of maximal infrarenal aortic diameters measured ex vivo (G). Student's *t‐*test, *n* = 6 per group, *p <  0.05. H,I) Representative western blotting (H) and quantification (I) of HASMCs infected with Ad‐null and Ad‐*ODC1* for 48 h. *n* = 6 per group. Student's *t‐*test, **p <  0.01, **p <  0.001. J) Putrescine concentrations of HASMCs infected with Ad‐null and Ad‐*ODC1* for 48 h. *n* = 3 per group, **p < 0.01. K–M) Twenty‐eight‐day infusion of Ang II (1000 ng/min/kg) in 4‐month‐old ApoE^−/−^ male mice challenged with or without 0.5% DFMO in drinking water. *n* = 16 per group. The timeline of DFMO feeding and Ang II infusion (K). Representative morphology of the aortas (L). The quantification of maximal suprarenal aortic diameters measured ex vivo, the incidence of AAA, and the death rate of mice. The red part in a histogram is the incidence and death rate of AAA model mice. The blue part in a histogram is the non‐disease and survival rate of AAA model mice (M). *n* = 16 per group. Student's *t‐*test, **p <  0.01.

Ornithine decarboxylase 1 (ODC1) is the rate‐limiting enzyme in the synthesis of polyamines that convert ornithine into putrescine.^[^ ^16^ ^]^ Higher levels of ODC1 were found in the aortas of C57BL/6J mice 14 days post‐perfusion with elastase than those in saline‐treated mice (**Figure** **4**A,B). Higher levels of ODC1 were also observed in HASMCs stimulated with Ang II, compared to the control groups (Figure 4C,D). To explore the role of ODC1, arteries were infected with either adenovirus (Ad)‐*ODC1* to induce ODC1 expression, or Ad‐Null, by smearing adenovirus mixed with 30% pluronic gel perivascularly after perfusion of the aortas with elastase (Figure S3A,B, Supporting Information). In the Ad‐*ODC1* group, the maximal infrarenal aortic diameters were larger and the amount of fragmented elastin was greater than in the Ad‐Null group (Figure 4E–G; Figure S3C,D, Supporting Information). The expression of *α*‐smooth muscle actin, smooth muscle 22, and calponin was decreased in HASMCs infected with Ad‐*ODC1* compared to that in HASMCs infected with Ad‐Null (Figure 4H,I; Figure S3E, Supporting Information). Putrescine levels in Ad‐*ODC1*‐infected HASMCs were higher than those in Ad‐Null‐infected HASMCs (Figure 3J).

Difluoromethylornithine (DFMO), also known as eflornithine, is an ODC1 inhibitor. DFMO has been approved for the treatment of African trypanosomiasis over the last 50 years.^[^ ^17^ ^]^ It has also been used to reduce hair growth and has a low rate of adverse events.^[^ ^18^ ^]^ In recent years, DFMO has been shown to have an inhibitory effect on many cancers^[^ ^19^ ^]^ and has been investigated in clinical trials for neuroblastoma (NCT02679144) and medulloblastoma (NCT04696029). We hypothesized that DFMO could rescue AAA by inhibiting ODC1. Four‐month‐old male ApoE^−/−^ mice were challenged with 0.5% DFMO in drinking water, followed 1 week later by Ang II infusion for 28 days. DFMO attenuated AAA, as shown by smaller maximal suprarenal aortic diameters and lower death rates in mice treated with DFMO compared to water‐administered mice (Figure 4K–M). Elastin fragmentation was also decreased in the aortic media from DFMO‐administered mice compared to that in water‐administered mice, as determined by EVG staining (Figure S3F,G, Supporting Information). In the elastase induction model, aortic diameters were significantly smaller in the DFMO group, with less elastin fragmentation compared to the control mice (Figure S3H–J, Supporting Information). Plasma putrescine concentrations were also lower in DFMO‐treated mice compared to the controls (Figure S3K, Supporting Information). In HASMCs; *α*‐smooth muscle actin, smooth muscle 22, and calponin were expressed at higher levels in DFMO‐treated cells than in control cells (Figure S3L,M, Supporting Information). These data demonstrate that the inhibition of ODC1 repressed AAA and that ODC1 exacerbates AAA by increasing putrescine levels (Figure S3N, Supporting Information).

### 2.5 VSMC‐GSDMD Stimulated ER Stress; CHOP Promoted Transcription of ODC1

**Figure 5:**
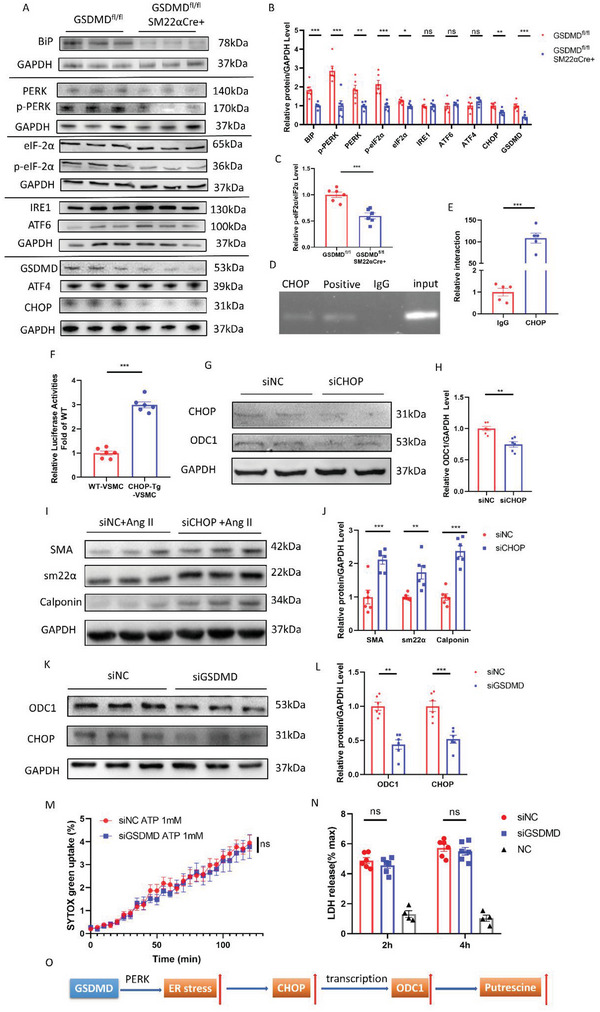
GSDMD stimulated PERK pathway and CHOP promoted transcription of ODC1. A,B) Representative western blotting (A) and quantification (B) of ER stress protein abundance from aortas of 14 days postperfusion of elastase. *n* = 6 per group. Student's *t‐*test, *p <  0.05, **p <  0.01, ***p <  0.001, ns: no significance. C) The quantification of the ratio of p‐eIF2*α*/eIF2*α*. Student's *t‐*test, ***p <  0.001. D) 293T cells were transfected with CHOP vector or empty vector. A chromatin immunoprecipitation assay was performed using an anti‐CHOP antibody with subsequent identification of ODC1 and GAPDH promoter sequences. E) ChIP DNA was used to perform q‐PCR with ODC1 promoter primers. *n* = 5 per group. Student's *t‐*test, ***p <  0.001. F) ODC1 activity in WT and *CHOP*‐Tg 293T cells; ODC1 promoter activity was measured by a dual‐luciferase reporter system. *n* = 5 per group. Student's *t‐*test, ***p <  0.001. G,H) Representative western blotting (G) and quantification (H) of ODC1 and CHOP from HASMCs transfected with CHOP siRNA or NC siRNA for 48 h. *n* = 6 per group. Student's *t‐*test, **p <  0.01. I,J) Representative western blotting (I) and quantification (J) of HASMCs stimulated with 100 nm Ang II for 24 h after transfecting with CHOP siRNA or NC siRNA for 24 h. *n* = 6 per group. Student's *t‐*test, *p <  0.05, ***p <  0.001. K,L) Representative western blotting (K) and quantification (L) of ODC1 and CHOP from HASMCs transfected with GSDMD siRNA or NC siRNA for 48 h. *n* = 6 per group. Student's *t*‐test, **p <  0.01, ***p <  0.001. M,N) LPS/ATP‐activated HASMCs transfected with or without GSDMD siRNA showing a time course of SYTOX green uptake (M) and LDH release (N) after 2 and 4 h. O) Schematic diagram of GSDMD‐PERK‐CHOP‐ODC1 axis.

ODC1 expression is controlled by many transcription factors, as shown in the Cistrome database.^[^ ^20^ ^]^ Among these, CHOP, a marker of ER stress, was of particular interest because mitigated ER stress was observed in HASMCs with GSDMD knockdown. ATF4, another downstream transcription factor of the PERK pathway, was not downregulated in mice with GSDMD deficiency (**Figure** **5**A–C; Figure S4A,B, Supporting Information). In ER stress, three pathways are activated: protein kinase R‐like ER kinase (PERK),^[^ ^21^ ^]^ inositol requiring enzyme 1 (IRE1),^[^ ^22^ ^]^ and activating transcription factor 6 (ATF6)^[^ ^23^ ^]^ (Figure S4C, Supporting Information). Binding immunoglobulin protein (BiP), also known as glucose‐regulated protein 78 (GRP78), is a chaperone abundantly present in the ER. BiP levels are elevated under ER stress.^[^ ^24^ ^]^ In this study, only the PERK‐eukaryotic initiation factor 2*α* (eIF2*α*) pathway was significantly stimulated. ER stress, particularly the upregulation of CHOP, plays an important role in VSMCs degeneration and AAA progression.^[^ ^25^ ^]^ CHOP deficiency can suppress 3‐aminopropionitrile fumarate‐induced thoracic aortic aneurysm/dissection.^[^ ^26^ ^]^ On this basis, we hypothesized that GSDMD could cause ER stress in VSMCs, upregulate CHOP, and promote the transcription of ODC1. A chromatin immunoprecipitation (ChIP) assay confirmed that CHOP could bind to the ODC1 promoter (Figure 5D,E). In a dual‐luciferase reporter assay performed to validate this result, CHOP overexpression resulted in increased activation of the ODC1 promoter compared with the vector control (Figure 5F). In HASMCs transfected with CHOP siRNA, ODC1 was downregulated at both the protein and mRNA levels (Figure 5G,H; Figure S4D, Supporting Information). Alpha‐smooth muscle actin, smooth muscle 22, and calponin were highly expressed in the CHOP siRNA group compared to the control group (Figure 5I,J), and putrescine was expressed at lower levels in CHOP siRNA‐transfected HASMCs (Figure S4E, Supporting Information). In addition, GSDMD knockdown reduced the levels of ODC1 and CHOP in HASMCs (Figure 5K,L). Transmission electron microscopy was used to identify any histological abnormalities in VSMCs. VSMCs from GSDMD^fl/fl^SM22Cre^+/−^ mice exhibited less aberrant ER morphology (e.g., reduced dilation of the ER intermembrane space) than those from GSDMD^fl/fl^ mice (Figure S4F, Supporting Information). The level of N‐GSDMD in the ER of HASMCs treated with Ang II was increased compared to that in the controls (Figure S4G, Supporting Information).

There are three ER stress inducers widely used: tunicamycin, which blocks the initial step of glycoprotein synthesis and thus inhibits the synthesis of N‐linked glycoproteins;^[^ ^27^ ^]^ thapsigargin (TG), which elicits ER stress through depletion of calcium and accumulation of unfolded or misfolded proteins in the ER;^[^ ^28^ ^]^ and the reducing agent dithiothreitol, which interferes with protein folding by blocking disulfide bond formation.^[^ ^29^ ^]^ Here, we used TG to induce a typical ER stress state. Compared to HASMCs treated with Ang II alone, putrescine concentrations were higher in HASMCs treated with both Ang II and TG (Figure S4H, Supporting Information). To rescue HASMCs from the GSDMD overexpression‐induced phenotype, we used *GSDMD*‐transgenic (*GSDMD*‐Tg) HASMCs with high GSDMD expression and tauroursodeoxycholic acid (TUDCA), a molecular chaperone suppressing ER stress.^[^ ^30^ ^]^ Contractile proteins were upregulated in *GSDMD*‐Tg HASMCs treated with TUDCA relative to TUDCA‐untreated *GSDMD‐*Tg cells (Figure S4I,J, Supporting Information). Collectively, these results demonstrate that GSDMD deficiency could alleviate ER stress through the PERK pathway and that the transcription factor CHOP could promote ODC1 expression.

As GSDMD is an essential mediator of pyroptosis and the N‐terminal fragment of GSDMD can form or promote the formation of a plasma membrane pore, we investigated whether GSDMD induced pyroptosis in HASMCs. We observed no alteration in lactate dehydrogenase (LDH) release or SYTOX green uptake in lipopolysaccharide‐primed HASMCs stimulated with ATP and treated with GSDMD siRNA or control siRNA (Figure 5M,N; Figure S4K, Supporting Information). These data indicate that GSDMD affected HASMC activity primarily through non‐pyroptosis‐mediated mechanisms (Figure 5O).

### 2.6 Putrescine Caused Phenotype Switching of VSMCs and Aggravated Inflammation

**Figure 6:**
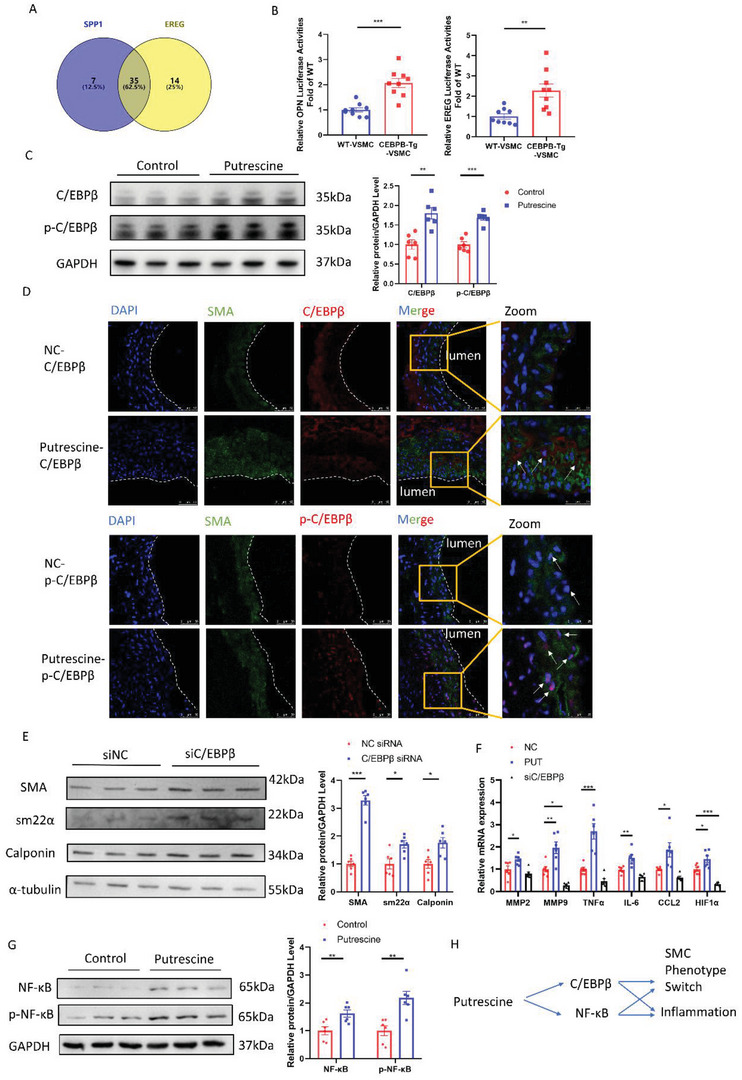
Putrescine switched the phenotype of VSMCs and aggravated inflammation. A) Venn diagram illustrating the predictive transcription factors of several classical genes for a VSMCs contractile phenotype via PROMO. B) OPN and EREG activity in WT and *CEBPB*‐Tg 293T cells; OPN and EREG promoter activity was measured by a dual‐luciferase reporter system. *n* = 9 per group. Student's *t*‐test, **p <  0.01, ***p <  0.001. C) Representative western blotting and quantification of C/EBP*β* and p‐C/EBP*β* from HASMCs incubated with or without 50 µm putrescine for 24 h. *n* = 6 per group. Student's *t*‐test, *p <  0.05, ***p <  0.001. D) Representative immunofluorescence staining of C/EBP*β* or p‐C/EBP*β* (red) and SM22*α* (green) in 14‐day postperfusion of elastase in 3‐month‐old C57BL/6J male mice challenged with or without 1% putrescine in drinking water, scale bar = 50 µm. E) Representative western blotting and quantification from HASMCs stimulated with 50 µm putrescine for 24 h after transfecting with C/EBP*β* siRNA or NC siRNA for 24 h. *n* = 6 per group. Student's *t*‐test, *p <  0.05, ***p <  0.001. F) mRNA abundance of inflammation factors in HASMCs incubated with 50 µm putrescine, C/EBP*β* siRNA, or NC siRNA for 24 h. *n* = 6 per group. One‐way ANOVA and Dunn post hoc test, *p <  0.05, **p <  0.01, ***p <  0.001. G) Representative western blotting and quantification of NF‐*κ*B and p‐NF‐*κ*B from HASMCs incubated with or without 50 µm putrescine for 24 h. *n* = 6 per group. Student's *t*‐test, **p <  0.01. H) Schematic diagram of putrescine affection on HASMCs.

To further confirm the role of putrescine in regulating the synthetic phenotype of HASMCs, we used the PROMO database^[^ ^31^ ^]^ to identify transcription factors regulating osteopontin (OPN) and epiregulin (EREG), two classical proteins of synthetic HASMCs that have been used as markers as reported previously^[^ ^32^ ^]^ (**Figure** **6**A). Among the 35 transcription factors putatively transcribing both OPN and EREG, CCAAT/enhancer‐binding protein *β* (C/EBP*β*) was predicted to be the most important. Based on this analysis, we postulated that putrescine may promote a switch of VSMC from the contractile to synthetic phenotype via C/EBP*β*. In support of this theory, experiments using the dual‐luciferase reporter system confirmed that OPN and EREG promoter activity increased in 293T cells overexpressing C/EBP*β* (Figure 6B). Furthermore, upregulation and increased phosphorylation of C/EBP*β* were observed in putrescine‐treated HASMCs (Figure 6C; Figure S4L, Supporting Information). In the elastase induction model, immunofluorescence staining of mouse aortas also showed increased C/EBP*β* expression and phosphorylation in VSMCs from putrescine‐treated mice compared to those from control mice (Figure 6D). In putrescine‐treated HASMCs, the expression levels of *α*‐smooth muscle actin, smooth muscle 22, and calponin were higher in the C/EBP*β* siRNA‐treated group compared to that in the control group (Figure 6E). Putrescine treatment and C/EBP*β* deletion also altered the expression of inflammatory factors in VSMCs. We detected increased expression of mRNAs encoding inflammatory factors (MMP2, MMP9, TNF*α*, IL‐6, CCL2, and HIF1a) after putrescine treatment and decreased expression of these factors in C/EBP*β* siRNA‐treated HASMCs (Figure 6F). Increased phosphorylation of nuclear factor‐kappa B (NF‐*κ*B), one of the 35 factors putatively involved in the transcription of both OPN and EREG, was observed in putrescine‐treated HASMCs (Figure 6G; Figure S4M, Supporting Information). As C/EBP*β* and NF‐*κ*B are the iconic transcription factors of senescence_,_^[^ ^33^ ^]^ we investigated whether the senescence‐associated secretory phenotype (SASP) was activated in putrescine‐treated HASMCs. The p16 level and mRNA levels of SASP‐related markers were detected in putrescine‐treated HASMCs and untreated HASMCs,^[^ ^34^ ^]^ and no significant difference was found (Figure S5A–C, Supporting Information). At the same time, *β*‐galactosidase staining indicated that putrescine treatment did not aggravate cell senescence (Figure S5D,E, Supporting Information). These data indicate that putrescine did not trigger senescence in VSMCs. Yes‐associated protein (YAP) promotes latent transforming growth factor‐*β* binding protein 4 (LTBP4)‐mediated elastic fibril assembly in VSMCs, which mitigates the formation of AAA.^[^ ^35^ ^]^ It was found that the levels of YAP and p‐YAP were decreased in putrescine‐treated HASMCs (Figure S5F,G, Supporting Information). These data indicated that putrescine may also aggravate AAA by promoting fibrillar ECM degradation. Overall, putrescine affected the switch of VSMCs from contractile to synthetic phenotype and increased HASMC inflammation via the C/EBP*β* and NF‐*κ*B pathways (Figure 6H).

## 3 Discussion

**Figure 7:**
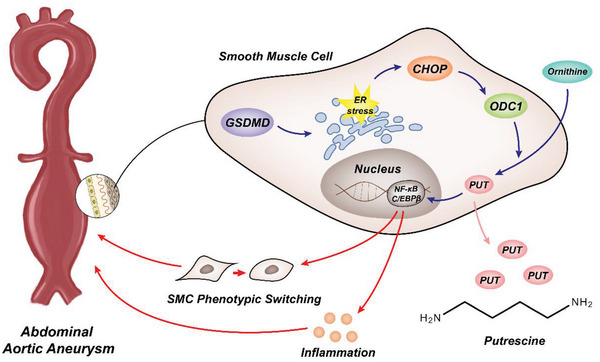
Schematic diagram of the potential mechanism by which GSDMD deficiency confers putrescine upregulated, VSMCs phenotype switching, and exacerbation of AAA.

We found that GSDMD was upregulated in both mouse models and patients with AAA. VSMC‐specific deletion of GSDMD alleviated AAA development. Our mechanistic studies revealed that GSDMD activates the ER stress pathway and upregulates CHOP, a transcription factor that regulates ODC1 expression. Elevated ODC1 increases the level of putrescine, which can upregulate both the total and phosphorylated levels of C/EBP*β*. Consequently, the expression of several synthetic proteins increased; promoting AAA (**Figure** **7**).

The expression of GSDMD in the aorta is associated with AAA formation. GSDMD releases inflammatory cytokines, such as interleukin (IL)‐1*β* and IL‐18^[^ ^36^ ^]^ and has been reported in many cardiovascular diseases such as atherosclerosis,^[^ ^37^ ^]^ ischemic heart disease,^[^ ^38^ ^]^ and diabetic cardiomyopathy.^[^ ^39^ ^]^ To date, numerous studies have been conducted on GSDMD in macrophages; however, the role of GSDMD in VSMCs, which are important components of vessels, has not yet been elucidated. According to the single‐cell transcriptome results from Luo et al.,^[^ ^11^ ^]^ GSDMD was significantly increased in the VSMCs of Ang II‐infused mice. In this study, we verified these results and found that GSDMD was significantly upregulated in AAA tissues of humans and mice. We postulated that GSDMD may increase in AAA as an initiation factor.

To test this hypothesis, we confirmed that GSDMD aggravates AAA in VSMC‐restricted GSDMD deficient mice. GSDMD causes pyroptosis in immune cells but plays other roles in VSMCs. Therefore, we speculated that several proteins and metabolites were altered downstream of GSDMD, accelerating the progression of AAA. Using a metabolomics approach, putrescine was found to be the most downregulated metabolite in GSDMD deficient mice. Putrescine is a major polyamine, in addition to spermidine and spermine,^[^ ^40^ ^]^ and is produced from L‐ornithine by the rate‐limiting enzyme ornithine decarboxylase.^[^ ^41^ ^]^ No significant changes in spermidine or spermine levels were observed. It has been reported that spermidine, as the downstream product of putrescine, suppresses the development of AAA, which also supports our hypothesis.^[^ ^42^ ^]^ Putrescine is essential for cell proliferation^[^ ^43^ ^]^ and is upregulated in many diseases, such as malignant squamous cell carcinomas,^[^ ^44^ ^]^ aging,^[^ ^45^ ^]^ and pulmonary hypertension.^[^ ^46^ ^]^ In our study, putrescine levels were upregulated in both the plasma and aorta of patients with AAA, suggesting that AAA was caused by high putrescine levels. AAA was significantly aggravated in mice treated with putrescine. We verified our hypothesis by administering putrescine to GSDMD deficient mice. Despite the GSDMD deficiency, putrescine could still promote AAA formation, indicating that GSDMD was upstream of putrescine. For the upstream of putrescine, we found that both ornithine and arginine were upregulated in the dataset of our previous study.^[^ ^47^ ^]^ However, only ornithine was significantly upregulated. So, there may be other factors in AAA that affect the metabolism of ornithine and arginine. Therefore, our results show that putrescine is upregulated by GSDMD; this may be a key mechanism by which GSDMD accelerates AAA development or progression.

The most critical enzyme in the production of putrescine is ODC1, a therapeutic target for many common diseases, such as gliomas and hepatitis B virus.^[^ ^48^ ^]^ Using an adenovirus encoding ODC1, we used an elastase induction model and overexpressed ODC1 perivascularly in the aorta. We found that ODC1 overexpression accelerated AAA development. DFMO is an irreversible inhibitor of ODC that is already used to combat cancers, including neuroblastoma, human acute T lymphoblastic leukemia, and malignant glioma.^[^ ^49^ ^]^ DFMO has also been used in the management of cardiovascular diseases, such as attenuating isoproterenol‐induced cardiac hypertrophy and protecting the blood–brain barrier.^[^ ^50^ ^]^ However, there are few reports of DFMO in the aorta. Feeding DFMO to AAA model ApoE^−/−^ mice resulted in a reduction in AAA, suggesting that it may have potential clinical value in the prevention and treatment of AAA. DFMO is well tolerated and few adverse events have been reported in clinical use;^[^ ^51^ ^]^ therefore, it could be repurposed for AAA treatment.

ER stress was downregulated in GSDMD‐deficient HASMCs. ER stress is caused by the accumulation of misfolded proteins. To restore protein folding capacity, a process called the unfolded protein response (UPR) is initiated.^[^ ^52^ ^]^ PERK is one of three proteins (IRE1, PERK, and ATF6) activated by UPR. In this study, the PERK‐eIF2*α* pathway was the only pathway that was significantly activated. It was reported that only the PERK pathway could be activated when ER stress occurred and Bip was downregulated in certain conditions.^[^ ^53^ ^]^ Therefore, it is possible that GSDMD selectively upregulated the level of PERK during this particular type of ER stress. CHOP is upregulated when these three proteins are activated by severe ER stress, leading to the upregulation of apoptosis. CHOP not only induces cell apoptosis by changing the expression of proteins, such as telomere repeat‐binding factor 3 and B‐cell lymphoma‐2,^[^ ^54^ ^]^ but also affects the synthesis of multiple proteins as a transcription factor.^[^ ^55^ ^]^ We investigated transcription factors upstream of ODC1 and found multiple factors in the CHIP database,^[^ ^20^ ^]^ including MYC and ZBP‐89.^[^ ^56^ ^]^ It has been reported that ER stress contributes to AAD via VSMC apoptosis, and CHOP deficiency could prevent the development of AAD.^[^ ^26^ ^]^ Suppression of ER stress was observed in GSDMD‐deficient HASMCs, and CHOP expression also decreased significantly. We hypothesized that GSDMD would induce ER stress, and consequently, CHOP expression. The transcription factor CHOP upregulates ODC1. Interestingly, Huang et al.^[^ ^57^ ^]^ showed that GSDMD could form pores in membranous organelles other than the plasma membrane, such as mitochondria. Therefore, the mechanism by which GSDMD causes ER stress may also involve the formation of membrane pores. This requires further studies using immunoelectron microscopy or other methods. Transmission electron microscopy results showed significant changes, such as dilated ER morphology, suggesting that ER‐related pathological processes occurred in the control group compared to the GSDMD‐deficient group.^[^ ^58^ ^]^ In summary, our work delineates the GSDMD–CHOP–ODC1 axis.

To determine the pathway responsible for the phenotype switching of VSMCs, we used the two proteins most representative of synthetic VSMCs: OPN and EREG, and predicted their transcription factors using the PROMO database. Among a large number of transcription factors, we selected C/EBP*β*, which is of great importance for OPN and EREG, as a hypothetical transcription factor. Both total and phosphorylated C/EBP*β* levels were upregulated in HASMCs treated with putrescine. Although the C/EBP*β* content in HASMCs was relatively low, the increase in phosphorylation was very significant. Furthermore, in immunofluorescence staining of aortas, phospho‐C/EBP*β* levels were significantly increased in the nuclei of VSMCs from putrescine‐treated AAA model mice. In addition, we demonstrated that inflammation was triggered by the increased level of inflammatory factors in putrescine‐treated HASMCs. Among the predicted factors, we chose to verify NF‐*κ*B as a representative transcription factor that aids inflammation. Total and phosphorylated NF‐*κ*B levels were increased in putrescine‐treated HASMCs; although, C/EBP*β* and NF‐*κ*B seemed similar to the case in SASP induction. However, senescence in putrescine‐treated HASMCs was not activated. Therefore, putrescine promotes AAA by simultaneously switching the VSMCs phenotype and aggravating inflammation.

The limitations of this study include the small size of the cohort. Our analysis was observational, and confounding variables such as all food and exercise could not be completely controlled. In addition, the ability of DFMO to reduce the incidence of AAA has not been verified in other populations. The study population only included patients diagnosed with AAA; therefore, our findings may not be applicable to patients with progressive AAA.

In summary, our current study revealed the destructive role of GSDMD and putrescine with respect to VSMCs phenotype switching and aggravation of AAA development. Therefore, our study suggests that GSDMD and putrescine suppression, similar to DFMO, may represent a novel therapy for AAA.

## 4 Experimental Section

### Human Subjects

This study was approved by the Institutional Review Board of Tongji Hospital (Tongji Medical College, Wuhan, China) and conducted in accordance with both the Declaration of Helsinki and the International Conference on Harmonization Guidelines for Good Clinical Practice. The experiments were carried out with the full, informed consent of the subjects. A total of 179 subjects were recruited from Tongji Hospital, Tongji Medical College, between January 2014 and September 2020, including 84 patients with AAA diagnosed by Doppler ultrasound or computed tomography of the aorta, and 95 age‐, sex‐, and risk factors‐ (including smoking, drinking, history of hypertension and diabetes) matched healthy control subjects with no significant systemic disease (ischemic heart disease, cancer, pulmonary disease, or infectious diseases). Peripheral blood samples were collected from all AAA patients and all healthy controls. Plasma was immediately separated by centrifugation and stored at −80 °C until analysis.

### Statistical Analysis

Statistical analyses were performed using GraphPad Prism 6.0 software (GraphPad Software, San Diego, CA, USA) and R (version 3.6.3; R Foundation, Vienna, Austria). The data shown are representative of two or three independent experiments. Continuous variables with normal distribution are expressed as means ± standard errors of the mean. Differences between the two groups were analyzed by Student's *t*‐test after the demonstration of homogeneity of variance with an F test. Comparisons between three groups were performed with a one‐way analysis of variance and Dunn's post hoc test. Continuous variables with non‐normal distributions are expressed as medians. Comparisons between groups were performed using the Mann–Whitney U test for nonparametric variables and Fisher's exact test or chi‐square test for categorical variables. The risk between log2‐transformed putrescine and AAA, and corresponding odds ratios, were analyzed using logistic regression in subgroup analyses. The receiver operator characteristic (ROC) curve was used to assess the diagnostic performance of putrescine to distinguish patients with AAA from healthy controls. A two‐sided *p*‐value less than 0.05 was used to define statistical significance.

## Conflict of Interest

The authors declare no conflict of interest.

## Author Contributions

J.G. and Y.C. contributed equally to this work. *Designed research studies*: J.G., Y.C., D.W.W., and L.Z. *Conducted experiments and analyzed data*: J.G., Y.C., H.W., X.L., K.L., and Y.X. *Provided reagents*: J.G., Y.C., X.X., Y.G., N.Y., X.Z., D.M., D.W.W., and L.Z. *Provided advice on manuscript writing*: H.S.L., Y.H.S., Y.L., J.Z., Y.E.C., A.D., D.W.W., and L.Z. *Wrote the manuscript*: J.G. and Y.C.

## Supporting information

Supporting Information

Supplemental Table 1

Supplemental Table 2

## Data Availability

The data that support the findings of this study are available from the corresponding author upon reasonable request.
