# Supplementary material for: Gasdermin D Deficiency in Vascular Smooth Muscle Cells Ameliorates Abdominal Aortic Aneurysm Through Reducing Putrescine Synthesis
Source: Adv Sci (Weinh). 2022 Dec 25;10(5):2204038. doi: 10.1002/advs.202204038 (PMC9929270; doi:10.1002/advs.202204038)
Supplement: Supplementary file 1 — Supporting Information [file ADVS-10-2204038-s002.pdf]

Supplemental online methods

Untargeted metabolomics

Untargeted metabolomics analysis was performed using a Q Exactive HFX orbitrap (Thermo, CA). Supernatant of tissue pulverized in 80% HPLC-grade methanol (1 μL) was loaded on a normal phase chromatography column, and eluted from the Orbitrap mass spectrometer with 50% ACN containing 10 mM ammonium acetate (pH 9) as eluent. Data with mass ranges of m/z 80-1200 were acquired at negative ion mode with data dependent MS-MS acquisition. The full scan and fragment spectra were collected with resolution of 70,000 and 17,500 respectively. The source parameters are as follows: spray voltage: 3000 v; heater temperature: 300 ^{o}C; capillary temperature: 320 ^{o}C; sheath gas flow rate: 35; auxiliary gas flow rate: 10. Metabolite identification was based on Tracefinder search with home-built database.

Human aortic samples

Human aortic samples were collected following patient consent according to a protocol approved by the Human Tissue Research Committee of Hebei Medical University and the study was conducted in accordance with the Declaration of Helsinki and the International Conference on Harmonization Guidelines for Good Clinical Practice. Informed consent was obtained from all volunteers. Abdominal aortic aneurysm (AAA) tissue specimens obtained in surgery were collected from patients with AAA (n=7). Normal control infrarenal aortic wall tissue specimens were obtained from organ donors (n=5).

Animal experiments

All animal experiments were performed according to the regulations approved by the Peking University Institutional Animal Care and Use Committee. Male ApoE$^{-/-}$ mice received water with or without 1% (wt/vol) putrescine at 13–15 weeks of age. One week later, mice were infused with angiotensin II (Ang II 1,000 ng/kg/min) using mini-osmotic pumps (Alzet Model 2004, DURECT Corporation) for 28 days to induce AAA. For difluoromethylornithine (DFMO), male ApoE$^{-/-}$ mice received water with or without 0.5% (wt/vol) DFMO at 13–15 weeks of age. One week later, mice were infused with Ang II (1,000 ng/kg/min) using mini-osmotic pumps (Alzet 2004) for 28 days to induce AAA.

GSDMD-flox (GSDMD$^{fl/fl}$) were obtained from Prof. Guotao Lu, who made this mouse model in Nanjing Biomedical Research Institute of Nanjing University. Female GSDMD$^{fl/fl}$ were crossed with male SM22Cre transgenic mice to generate GSDMD SMC-deleted mice (GSDMD$^{fl/fl}$SM22Cre$^{+/-}$). All study mice were bred to the ApoE$^{-/-}$ background. GSDMD$^{fl/fl}$SM22Cre$^{+/-}$ ApoE$^{-/-}$ and GSDMD$^{fl/fl}$ ApoE$^{-/-}$ littermates were infused with Ang II (1,000 ng/kg/min) for 28 days at 14–16 weeks of age. After 28 days of AngII infusion, animals were euthanized, and their aortas were harvested and fixed in 10% formalin or 4% paraformaldehyde. After removing adventitia, we measured the maximal outer diameter (*ex vivo*) of the suprarenal region.

Elastase-induced AAA model was performed as described previously[1]. The operation was accomplished under general anesthesia. The infrarenal region of abdominal aorta was exposed. A gauze with 40 μl of elastase was placed around the aorta for 40 min. A gauze with 40 μl of 0.9% NaCl place around the aorta for 40 min was used as a control. After removal of the gauze, the surgical site with cleaned with 0.9% NaCl. After 14 days, all animals were euthanatized and

aortas were harvested, fixed, and cleaned for maximal outer diameter measurements in the infrarenal region. Image J was used to measure the maximum outer diameter of aortas.

Mass Spectrometry

For plasma samples, fasting blood samples were collected into K2 EDTA vacutainer tubes and immediately placed in a refrigerator (4 °C). Samples were centrifuged within 2 h at 3000 g for 10 min at 4 °C. Supernatants (plasma) were separated and transferred into new vials, and frozen (-80 °C) until preparation. Frozen plasma samples were thawed at 4 °C. A total of 100 μL of plasma was spiked with 5 μL of putrescine (13C4) (5 μM), followed by addition of 300 μL of methanol. After vortex mixing for 4 min and centrifuging at 14,000 rpm for 4 min at 4°C, the supernatant was separated, then add a certain volume of acidic water to the supernatant, mix well and wait for loading the sample.

For tissue samples, frozen tissue with the same wet weight was accurately weighed into a 2 mL homogenization tube containing four ceramic beads (3.0 mm diameter). Pre-cooled extraction solvents (500uL of 80%(v/v) HPLC-grade methanol, which contains 5 μM of putrescine (13C4), were added, and the tissue was homogenized three times for 30 s at a shock velocity of 4.0 m/s using a high-throughput tissue homogenizer. After homogenization, samples were centrifuged at 14,000 rpm for 4 min at 4°C. Supernatant were separated, then acidic water was addedto the supernatant and mixed well.

All the above mixed supernatant was then passed through solid-phase extraction (SPE) cartridges (Oasis MCX 3cc cartridges, 60 mg, Waters, Milford, MA, USA). The sorbent was conditioned with 3 mL methanol and equilibrated with 3 mL of water before samples were

applied. After supernatants were eluted, 2 mL water and 2 mL methanol were passed through cartridges, and then eluted with 2 ml ammoniated methanol, the eluate was collected and dried using a Savant concentrator with RVT-5105 refrigerated vapor. Before analysis, the extracts were resuspended in 100 ul of 0.5% FA/water (v/v). A 5 μL aliquot of sample was injected into the LC-MS system for analysis.

The chromatography was performed on a Shimadzu Prominence system with a binary pump, an online degasser, an autosampler and a column oven (Shimadzu Scientific Instruments, INC., Columbia, MD, USA). The separation was achieved on a Waters ACQUITY HSS T3 column (2.1 × 100 mm, 1.8 μm) at 40°C. The gradient elution was programmed with the mobile phase consisting of formic acid in 0.5% (vol/vol) water (A) and 0.1% (vol/vol) formic acid in acetonitrile (B) at a flow rate of 0.3 mL/min as follows: 0–1.5min, 5–100% B (vol/vol); 1.5–3min, 100% B. The system was returned to the initial conditions in 0.1 min and re-equilibrated for 3min. The autosampler temperature was set at 4°C and the injection volume was 5μL.

Mass spectrometric detection was performed on a SCIEX QTRAP 6500+ system (SCIEX, Foster City, CA, USA) with an electrospray ionization source in positive ion mode. Putrescine was monitored in multiple reaction monitoring mode using the precursor-to-product ion transitions of m/z 89.0 →72.1 for putrescine, m/z 93.2 →76.2 for putrescine (13C4).

Declustering potentials and collision energy were 28 V and 13 eV for putrescine, 15 V and 11 eV for IS, respectively. Collision exit potential were 9 V for all analytes. The mass spectrometer was operated under the following optimum parameters: curtain gas (nitrogen), 30; ionspray voltage, 5500 V; source temperature, 550°C; ion source gas 1 (zero-grade air), 55; ion source gas 2 (zero-grade air), 60; and collision gas (nitrogen), LOW. Analyst software 1.7.3 (Sciex)

was used for the data acquisition as well as processing.

Histology

Aortas were fixed in 4% paraformaldehyde overnight. After dehydration with 20% sucrose, aortas were embedded in optimal cutting temperature compound and sliced with a cryostat with 7μm tissue sections. Sections were stained with Elastin Van Gieson (EVG) and hematoxylin-eosin (HE). Sections were imaged using a BX43 Olympus microscope (Japan). Representative histological photomicrographs are presented. For the grade of elastin degradation, the definition is as followed: grade 1, no degradation; grade 2, mild elastin degradation; grade 3, severe elastin degradation; and grade 4, aortic rupture.

Immunohistochemistry

Frozen sections were removed from a freezer and placed at room temperature for 30 minutes. The sections were immunostained using a rabbit anti-mouse GSDMD antibody (1:200 dilution, Cat#ab219800, abcam, UK). Secondary antibody used were biotinylated goat anti-rabbit IgG (Cat#PV9000, ZSGB-BIO, China). Negative controls included non-immune rabbit IgG2a, no primary antibody control, and no primary and secondary antibody control. Positive immunoreactivity was visualized as red color by oxidation of aminoethyl carbazole. Sections were imaged using a BX43 Olympus microscope.

Immunofluorescence

Frozen sections were removed from a freezer and placed at room temperature for 30 minutes.

Sections were incubated with primary antibodies at 4°C overnight, and then incubated with secondary antibodies for 60 min at room temperature. Isotype match non-immune IgG2a, omission of primary antibody, and omission of both primary and secondary antibodies were used as negative controls. Mounting medium with DAPI (ZSGB-BIO, ZLI-9557, China) was applied for nuclear staining. Sections were imaged through a Leica TCS SP8 STED confocal fluorescent microscope (3X, Germany).

Target antigen	Vendor or Source	Catalog #	Working concentration
Mouse GSDMD	abcam	ab219800	5 ug/ml
Mouse SMA	abcam	ab7817	5 ug/ml
Mouse C/EBPβ	abcam	ab15050	5 ug/ml
Mouse p-C/EBPβ	abcam	ab52194	5 ug/ml
DAPI	ZSGB-BIO	ZLI-9557	-
Alexa Fluor 488-conjugated goat anti-mouse IgG	abcam	ab150077	0.5 ug/ml
Alexa Fluor 555-conjugated goat anti-rabbit IgG	abcam	ab150077	0.5 ug/ml

Cell isolation and cell culture

Primary SMCs were isolated by collagenase digestion from mouse aortas as described previously[2]. Briefly, thoracic aortas were dissected by stripping away the adventitia and intima. Aortas were minced and incubated with 0.1% type I collagenase (SCR103, Sigma) and 0.1% trypsin for 10 min and freshly prepared 0.1% type I collagenase for 2-4 h at 37°C. SMCs were then collected from the buffer, and cultured in a SMC medium (SMCM, 1101, ScienCell Research Laboratories, USA) in the incubator at 37°C with 5% CO2. Cells from passages 3-5 were used for further experiments.

Human aortic smooth muscle cells (HASMCs) were obtained from ScienCell Research Laboratories (6110, USA). HASMCs was cultured in SMCM containing 2% fetal bovine serum (FBS) in a humidified incubator at 37°C with 5% CO_2. Before use, cell dishes were coated with poly-L-lysine (0403, ScienCell Research Laboratories, Carlsbad, CA, USA) for 1 h. Cells from passages 3-5 were used for further experiments.

Western Blot Analysis

HASMCs or aortas were washed with cold PBS and lysed in radio immunoprecipitation assay buffer to extract protein of whole cell. Equal amounts of total protein were resolved using 10% SDS-PAGE and transferred onto nitrocellulose membranes. After blocked in 5% non-fat milk for 1 h, the membranes were incubated with primary antibodies as follows. The proteins signal was visualized by the enhanced chemiluminescence system (ECL, Thermo, 32109).

Target antigen	Vendor or Source	Catalog #	Working concentration
Mouse GSDMD	abcam	ab209845	1 ug/ml

Human GSDMD	abcam	ab210070	1 ug/ml
Mouse/human SMA	abcam	ab7817	1 ug/ml
Mouse/human TAGLN	abcam	ab14106	1 ug/ml
Mouse/human Calponin	abcam	ab46794	1 ug/ml
Mouse/human ODC1	abcam	ab193338	1 ug/ml
Flag	abcam	ab205606	0.5 ug/ml
Mouse/human CHOP	CST	2895S	1 ug/ml
Mouse/human BIP	CST	3177S	1 ug/ml
Mouse/human PERK	Proteintech	24390-1-AP	1 ug/ml
Mouse/human p-PERK	CST	3179S	1 ug/ml
Mouse/human eIF-2α	Proteintech	11233-1-AP	1 ug/ml
Mouse/human Phospho-eIF2α-S51	Enogene	E2530745	1 ug/ml
Human Cleaved N-terminal GSDMD	abcam	ab215203	1 ug/ml
Mouse/human Calnexin	Santa Cruz	sc-23954	1 ug/ml
Mouse/human C/EBPβ	abcam	ab32358	1 ug/ml
Mouse/human Phospho-C/EBPβ	abcam	ab52194	2 ug/ml
Mouse/human NF-κB	CST	8242S	1 ug/ml
Mouse/human Phospho-NF-κB	CST	3033S	1 ug/ml

Human YAP	CST	14074T	1 ug/ml
Human Phospho-YAP	CST	13008T	1 ug/ml
Human Calmodulin	Abclonal	A4885	1 ug/ml
Mouse/human GAPDH	Proteintech	60004-1-Ig	0.5 ug/ml
Mouse/human α-tubulin	abcam	ab7291	0.5 ug/ml
Mouse/human β-actin	Proteintech	66009-1-Ig	0.5 ug/ml
HRP-conjugated goat anti mouse IgG	MBL	330	0.2 ug/ml
HRP-conjugated goat anti rabbit IgG	MBL	458	0.2 ug/ml

Luciferase Reporter Assays

HEK293T were transiently co-transfected with luciferase reporter and β-actin plasmids by Jet-PRIME reagent as described previously[3]. After 30 h, transfected cells were harvested for luciferase activity assay using a luciferase assay kit (Promega, Madison, WI). The transcriptional activity was indicated as the ratio of luciferase activity to respective β-actin activity.

RNA Extraction and Quantitative Real Time PCR

Total RNA was extracted from cultured HASMCs using the TRIzol (Invitrogen) according to the manufacturer's instructions. RNA concentrations were measured using Nanodrop 2000 (Invitrogen). RNA (1 μg) of each sample was used to synthesize cDNA using the first-strand

cDNA synthesis kit (TRANSGEN, China) according to the manufacturer's instructions. RT-PCR was performed using Platinum SYBR Green qPCR Super Mix (TRANSGEN, China), and the experiments were conducted on AriaMx Real-Time PCR system (Agilent Technologies). The primer sequences were as Table.

Gene name	Forward	Reverse
Ire-1	CATCCCCATGCCGAAGTTCA	CTGCTTCTCTCCGGTCAGGA
Atf6	AATATATGCTAGGGTTAGAGGC	TTCTCTGACACAACTTCATC
Atf4	GGCCAAGCACTTCAAACCTC	GAGAAGGCATCCTCCTTGCT
Xbp1	CTGAGTCCGCAGCAGGTG	GTCCAGAATGCCCAACAGGA
eIf2α	GCCTTTCTTGAACTCTCACC	CCGTGCTTTCTGTGAAGTGT
Perk	CAGTGGGATTTGGATGTGGG	GGAATGATCATCTTATTCCCAAA
Chop	ATGAGGACCTGCAAGAGGTCC	TCCTCCTCAGAGAGAGAGAAAA
MMP2	TACAGGATCATTGGCTACACACC	GGTCACATCGCTCCAGACT
MMP9	AGACCTGGGCAGATTCCAAAC	CGGCAAGTCTTCCGAGTAGT
TNFα	GAGGCCAAGCCCTGGTATG	CGGGCCGATTGATCTCAGC
IL-6	ACTCACCTCTTCAGAACGAATTG	CCATCTTTGGAAGGTTCAGGTTG
CCL2	CAGCCAGATGCAATCAATGCC	TGGAATCCTGAACCCACTTCT
HIF-1α	TGATTGGATCACCCGAGAAGG	CGGAAGACTATTGCAGTCCCT
β-actin	CATTGCTGACAGGATGCAGAAGG	TGCTGGAAGGTGGACAGTGAGG
Odc1-CHIP	GAATTTCCCACGCGGCTTTT	CGGATCCTGAGTCTCCAAGC

Small interfering RNA transfection

Small interfering RNA (siRNA) against GSDMD was 5′-CCCGUUAUAAGUGUGUCAATT-3‘, designed by GenePharma (Shanghai, China). siRNA against CHOP was obtained from Santa Cruz (sc-35437). siRNA against C/EBPβ was 5′-CCCUGAGUAAUCACUUAAATT-3′, described by Huang *et al*[4]. The scrambled siRNA served as a negative control. HASMCs cultured in 6-well plates were transfected with 20 nM siRNA per well through transfection reagent Jet PRIME for 4 h and then incubated in SMCM with 2% FBS for 24-48 h.

Recombinant adenovirus construction

Mouse-derived adenoviruses with tomato and 3flag tags were constructed by LiKeLi Company, Beijing. Ad5-Null was used as a negative control (NC). For *in vivo* experiments, a single exposure of 1×10^9 plaque forming units Ad5-ODC1 or Ad5-Null was mixed with 30% hydrogel pluronic F-127 (Sigma, USA) and applied perivascularly to the carotid artery. For *in vitro* experiments, Ad5-ODC1 or Ad5-Null was added to 6-well plates containing 50–70% confluent HASMCs for 4 h, and then the cells incubated in SMCM with 2% FBS for 48 h.

SYTOX green uptake

To assess lipopolysaccharide (LPS) and ATP-induced cell death, HASMCs was seeded in 96-well plates overnight and treated with 500 ng/ml LPS for 2h. And then added 2.5 μM SYTOX green and 1 mM ATP. Fluorescence at 528 nM after excitation at 485 nM was continually recorded every 5 min using a Clariostar Plus Microplate Reader (BMG LABTECH, German).

Percentage cytotoxicity was calculated based on maximum fluorescence from unstimulated cells lysed with 1% Triton X-100.

LDH release

HASMCs was pre-treated with 500 ng/ml LPS and stimulated with 1 mM ATP. After 2 or 4 h stimulation of HASMCs, supernatant was collected and lactate dehydrogenase (LDH) release was quantified using LDH Cytotoxicity Detection Kit (Takara, MK401, Japan) according to the manufacturer's instructions. Percentage cytotoxicity was calculated based on maximum LDH release from unstimulated cells lysed with 1% Triton X-100.

ChIP analysis

For chromatin immunoprecipitation (ChIP) analysis, chromatin of HASMCs with ODC1 promoter transfected was extracted by Chromatin Extraction Kit (ab117152, abcam, UK) according to the manufacturer's instructions. ChIP Kit - One Step (ab117138, abcam, UK) and CHOP antibody (2895S, CST, USA) was used to get the DNA that interacted with CHOP according to the manufacturer's instructions. Subsequently, the immunoprecipitated DNA was quantified by real time PCR. GAPDH was used as positive control.

ER isolation

For endoplasmic reticulum (ER) isolation, Minute ER Enrichment Kit (Invent, ER-036, USA) was used according to the manufacturer's instructions. The protein of ER was collected for further experiments.

Transmission Electron Microscopy

Smooth muscle cells were fixed with 2.5% glutaraldehyde for 1h at room temperature. Samples were fixed with 1% osmium tetroxide for 1h, dehydrated through ethanol, and embedded in Epon. The ultramicrotome (Leica EM UC6 C) was used to make ultrathin sections (70 nm). The sections mounted on copper grids were stained with uranyl acetate and lead citrate and then examined by the transmission electron microscopy system (Talos L120C).

Senescence-associated-β-galactosidase (SA-β-gal) staining

By using the kit of SA-β-gal (C0602, Beyotime Biotechnology, China), cultured cells were washed in ice-cold PBS for three times and then fixed for 10 min at room temperature. After washing for three times, the cells were incubated in staining buffer for 12-hours at 37℃. After the stain, the cell could keep in ice-cold PBS for a few days. SA-β-gal images were analyzed with Image J software. The average optical density of the senescence (blue) was evaluated.

Reference

1 Liu Z, Wang Q, Ren J *et al.* Murine abdominal aortic aneurysm model by orthotopic allograft transplantation of elastase-treated abdominal aorta. *J Vasc Surg* 2015; **62**:1607-1614 e1602.

2 Chamley-Campbell J, Campbell GR, Ross R. The smooth muscle cell in culture. *Physiol Rev* 1979; **59**:1-61.

3 Ma D, Zheng B, Suzuki T *et al.* Inhibition of KLF5-Myo9b-RhoA Pathway-Mediated Podosome

Formation in Macrophages Ameliorates Abdominal Aortic Aneurysm. *Circ Res* 2017; **120**:799-815.

4 Huang E, Huang H, Guan T *et al.* Involvement of C/EBPbeta-related signaling pathway in methamphetamine-induced neuronal autophagy and apoptosis. *Toxicol Lett* 2019; **312**:11-21.

Supplemental Figures

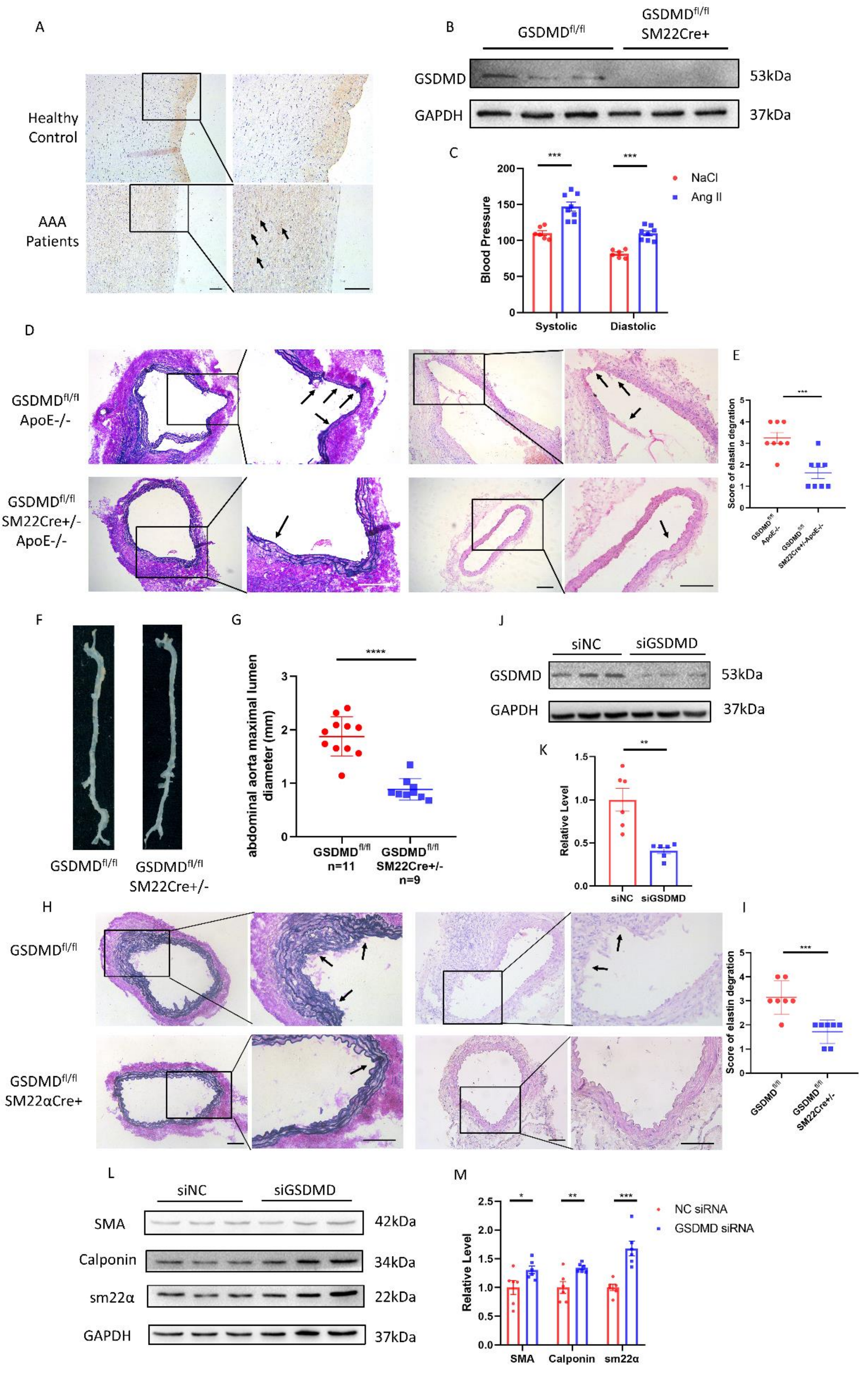

Supplemental Figure 1

A: Representative immunohistochemical staining of GSDMD in human aortas.

B: Representative western blotting of GSDMD in SMCs isolated from aortas of $GSDMD^{fl/fl}SM22Cre^{+/-}$ and $GSDMD^{fl/fl}$ mice.

C: The blood pressure of $GSDMD^{fl/fl}SM22Cre^{+/-}ApoE^{-/-}$ and $GSDMD^{fl/fl}ApoE^{-/-}$ mice infused with Ang II or saline for 28 days. n=6 for saline-treated group and n=8 for Ang II -treated group. Student's t test, ***$p < 0.001$.

D: Representative EVG staining and HE staining of suprarenal aortas. The black arrows indicate the fragmented elastin.

E: The grade of elastin degradation. n=8 per group. Student's t test, ***$p < 0.001$.

F-I: Fourteen-day postperfusion of elastase in three-month-old $GSDMD^{fl/fl}SM22Cre^{+/-}$ and $GSDMD^{fl/fl}$ male mice.

F: Representative morphology of the aortas.

G: The quantification of maximal infrarenal aortic diameters measured *ex vivo*. n=11 for $GSDMD^{fl/fl}$ group and n=9 for $GSDMD^{fl/fl}SM22Cre^{+/-}$ group. Student's t test, ***$p < 0.001$.

H: Representative EVG staining and HE staining of infrarenal aortas. The black arrows indicate fragmented elastin.

I: The grade of elastin degradation. n=7 per group. Student's t test, ***p < 0.001.

J-K: Representative western blotting (J) and quantification (K) of GSDMD from HASMCs transfected with GSDMD siRNA or NC siRNA for 48h. n=6 per group. Student's t test, **$p < 0.01$.

L-M: Representative western blotting (L) and quantification (M) from HASMCs transfected with GSDMD siRNA or NC siRNA for 48 h. n=6 per group. Student's t test, *$p < 0.05$, **$p < 0.01$, ***$p < 0.001$.

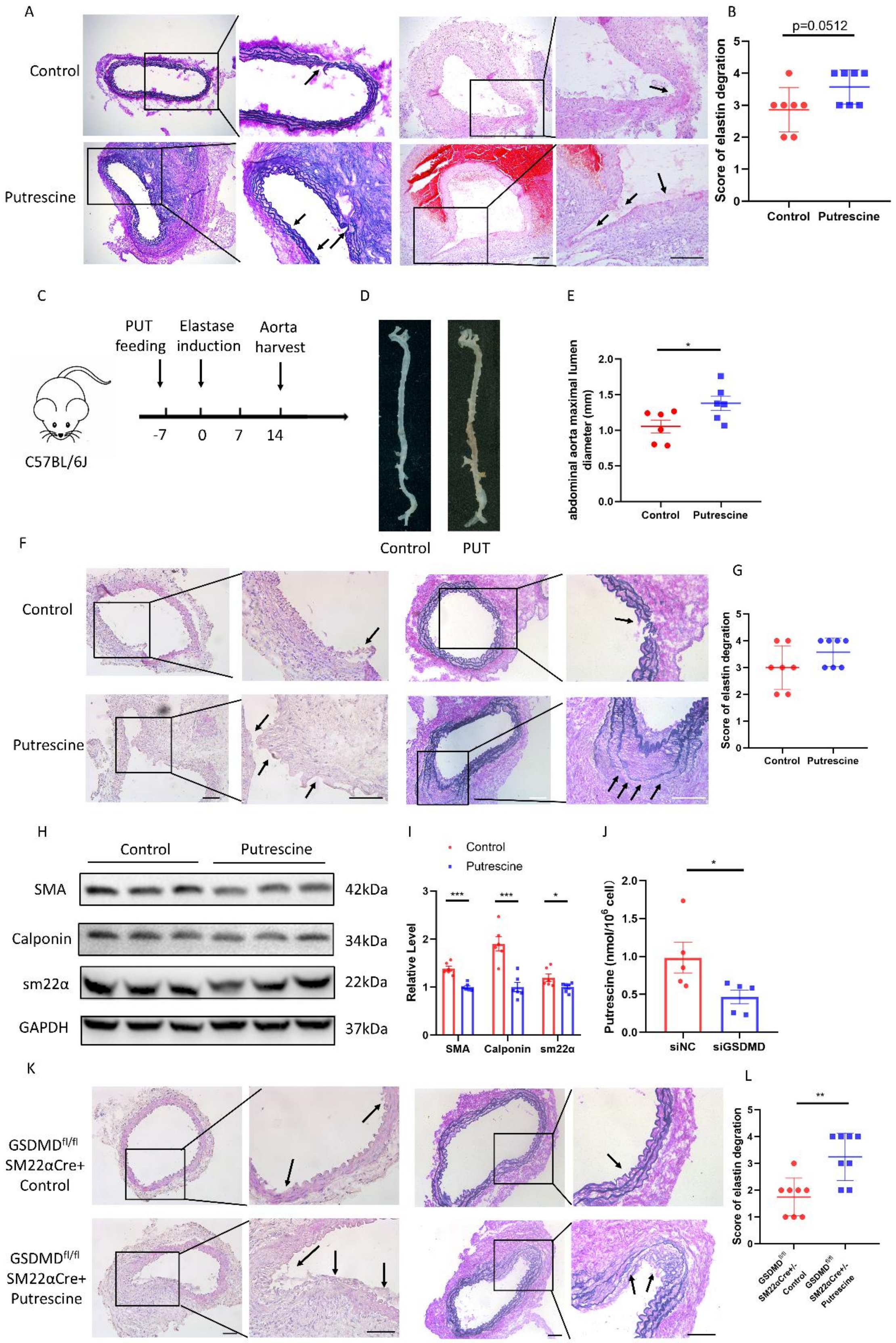

Supplemental Figure 2

A: Representative EVG staining and HE staining of suprarenal aortas. The black arrows

indicate the fragmented elastin.

B: The grade of elastin degradation. n=7 per group. Student's t test.

C-G: Fourteen-day postperfusion of elastase in three-month-old C57BL/6J male mice challenged with or without 1% putrescine in drinking water.

C: The timeline of putrescine feeding and elastase induction.

D: Representative morphology of the aortas.

E: The quantification of maximal infrarenal aortic diameters measured *ex vivo*. n=6 per group. Student's t test, $*p < 0.05$.

F: Representative EVG staining and HE staining of infrarenal aortas. The black arrows indicated fragmented elastin.

G: The grade of elastin degradation. n=7 per group. Student's t test.

H-I: Representative western blotting (H) and quantification (I) of HASMCs incubated with or without 50 μM putrescine for 24 h. n=6 per group. Student's t test, $*p < 0.05$, $***p < 0.001$.

J: Putrescine concentrations of HASMCs transfected with GSDMD siRNA or NC siRNA for 48 h. $*p < 0.05$.

K: Representative EVG staining and HE staining of infrarenal aortas. The black arrows indicated fragmented elastin.

L: The grade of elastin degradation. n=8 per group. Student's t test, $**p < 0.01$.

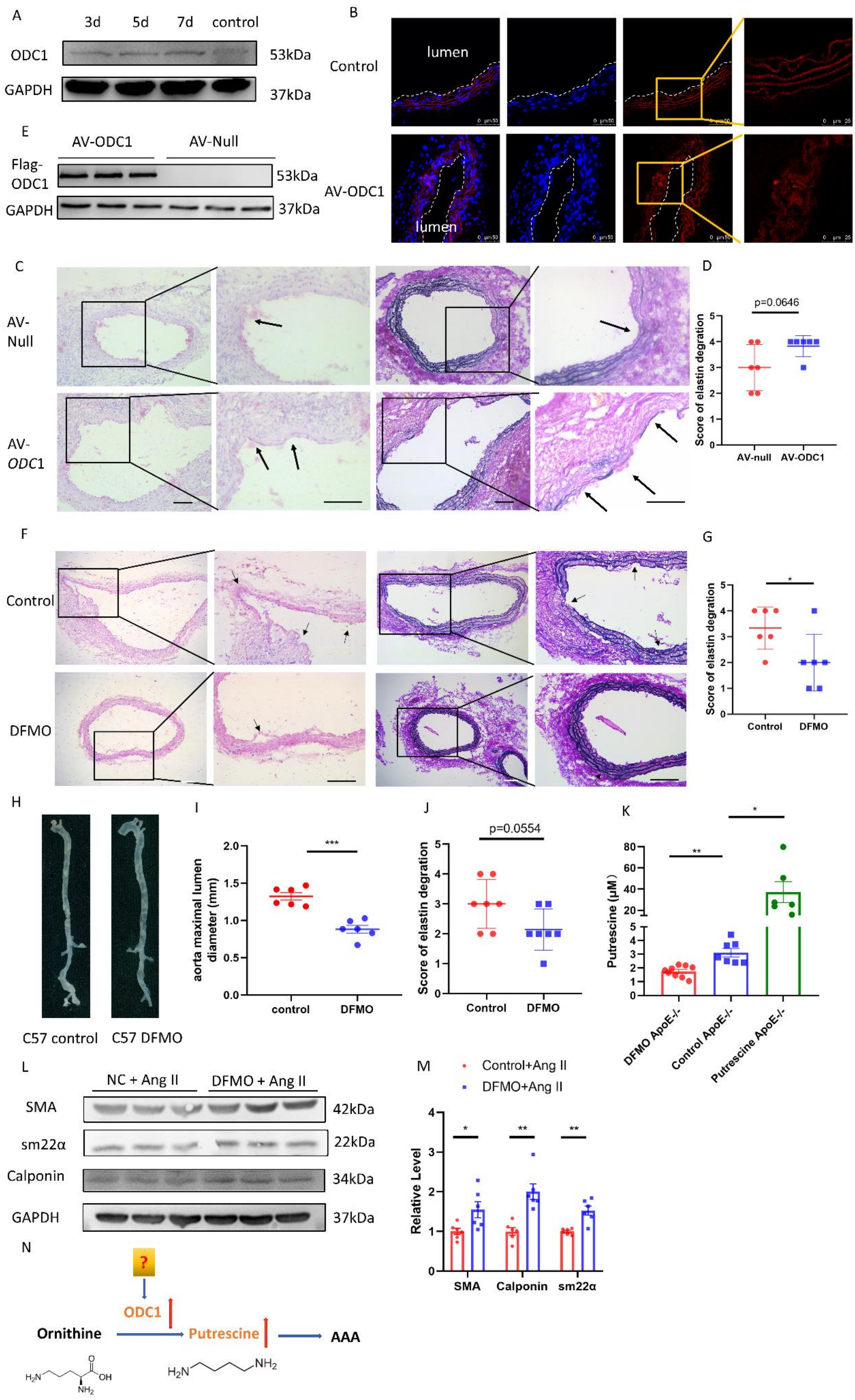
A
3d
5d
7d
control
ODC1
53kDa
GAPDH
37kDa
B
Control
lumen
AV-ODC1
lumen
E
AV-ODC1
AV-Null
Flag-ODC1
53kDa
GAPDH
37kDa
C
AV-Null
AV-ODC1
D
p=0.0646
Score of elastin degration
AV-null
AV-ODC1
F
Control
DFMO
G
Score of elastin degration
Control
DFMO
H
C57 control
C57 DFMO
I
aorta maximal lumen diameter (mm)
control
DFMO
J
p=0.0554
Score of elastin degration
Control
DFMO
K
Putrescine (μM)
DFMO ApoE-/-
Control ApoE-/-
Putrescine ApoE-/-
L
NC + Ang II
DFMO + Ang II
SMA
42kDa
sm22α
22kDa
Calponin
34kDa
GAPDH
37kDa
M
Control+Ang II
DFMO+Ang II
Relative Level
SMA
Calponin
sm22α
N
?
ODC1
Ornithine
Putrescine
AAA

Supplemental Figure 3

A: Western blotting of ODC1 from aortas of C57BL/6J male mice in which abdominal aortas periadventitially infected with Ad-Odc1 for 3/5/7 days.

B: Representative immunofluorescence staining of Ad-*ODC1* (red, with tdTomato tag) in control and periadventitially infected mice, scale bar=50 μm.

C: Representative EVG staining and HE staining of infrarenal aortas. The black arrows indicate the fragmented elastin.

D: The grade of elastin degradation. n=6 per group. Student's t test.

E: Western blotting of FLAG from HASMCs transfected with Ad-null or Ad-*ODC1* for 48 h.

F: Representative EVG staining and HE staining of suprarenal aortas. The black arrows indicated fragmented elastin.

G: The grade of elastin degradation. n=6 per group. Student's t test, *$p < 0.05$.

H-J: Fourteen-day postperfusion of elastase in three-month-old C57BL/6J male mice challenged with or without 0.5% putrescine in drinking water.

H: Representative morphology of the aortas.

I: The quantification of maximal infrarenal aortic diameters measured *ex vivo*. n=6 per group. Student's t test, ***$p < 0.001$.

J: The grade of elastin degradation of EVG staining of infrarenal aortas. n=7 per group. Student's t test.

K: Putrescine concentrations in plasma of mice challenged with DFMO or putrescine. n=9 for DFMO-treated group, n=7 for water-treated group and n=6 for putrescine-treated group. One-way ANOVA and Dunn post hoc test, *$p < 0.05$, **$p < 0.01$.

L-M: Representative western blotting (L) and quantification (M) from HASMCs incubated with or without 100 μM DFMO for 24 h and then incubated with 100 nM Ang II for 24 h. n=6 per group. Student's t test, *p < 0.05, **p < 0.01.

N: Schematic diagram of the impact of ODC1 on AAA.

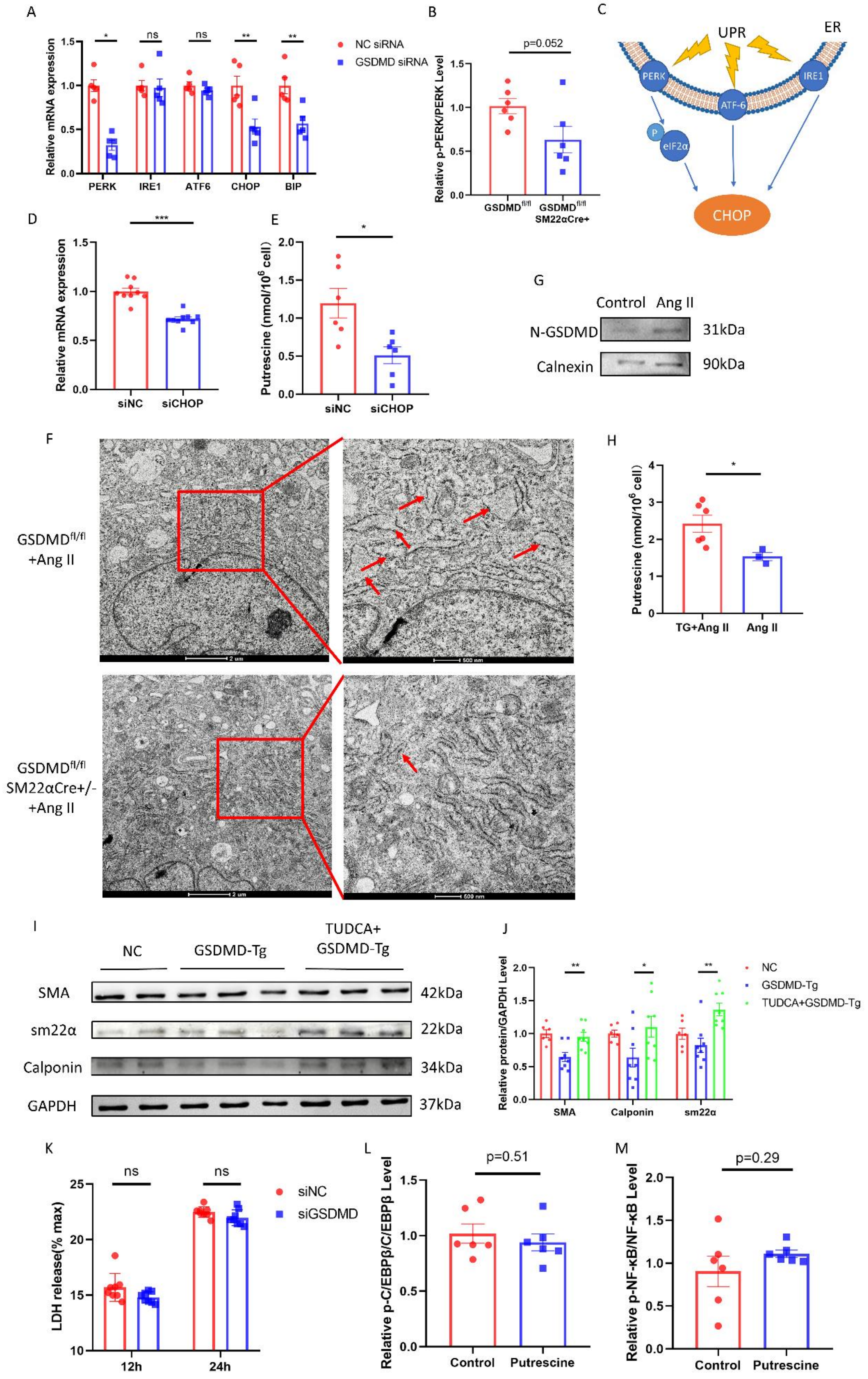

A
NC siRNA
GSDMD siRNA
Relative mRNA expression
PERK
IRE1
ATF6
CHOP
BIP
B
p=0.052
Relative p-PERK/PERK Level
C
UPR
ER
PERK
ATF-6
IRE1
eIF2α
CHOP
D
Relative mRNA expression
siNC
siCHOP
E
Putrescine (nmol/10^6 cell)
siNC
siCHOP
G
Control
Ang II
N-GSDMD
31kDa
Calnexin
90kDa
F
GSDMDfl/fl +Ang II
GSDMDfl/fl SM22αCre+/- +Ang II
H
Putrescine (nmol/10^6 cell)
TG+Ang II
Ang II
I
NC
GSDMD-Tg
TUDCA+ GSDMD-Tg
SMA
42kDa
sm22α
22kDa
Calponin
34kDa
GAPDH
37kDa
J
NC
GSDMD-Tg
TUDCA+GSDMD-Tg
Relative protein/GAPDH Level
SMA
Calponin
sm22α
K
siNC
siGSDMD
LDH release(% max)
12h
24h
L
p=0.51
Relative p-C/EBPβ/C/EBPβ Level
Control
Putrescine
M
p=0.29
Relative p-NF-κB/NF-κB Level
Control
Putrescine

Supplemental Figure 4

A: mRNA abundance of ER stress-related factors in HASMCs transfected with GSDMD siRNA or NC siRNA for 48 h. n=5 per group. Student's t test, *$p < 0.05$, **$p < 0.01$, ***$p < 0.001$, ns: no significance.

B: The quantification of the ratio of p-PERK/PERK. Student's t test.

C: Schematic diagram of the 3 pathways of ER stress.

D: mRNA expression of ODC1 in HASMCs transfected with CHOP siRNA or NC siRNA. n=9 per group. Student's t test, ***$p < 0.001$.

E: Putrescine concentrations of HASMCs transfected with CHOP siRNA or NC siRNA for 48 h. n=6 per group. Student's t test, *$p < 0.05$.

F: Transmission electron microscopy analysis was performed in SMCs isolated from GSDMD$^{fl/fl}$SM22Cre$^{+/-}$ and GSDMD$^{fl/fl}$ mice incubated with 100 nM Ang II for 24 h. The red arrows indicated the dilations of ER intermembrane spaces. Scale bar=2 µm & 50 nm.

G: Representative western blotting of N-GSDMD from the ER of HASMC incubated with 100 nM Ang II for 24 h.

H: Putrescine concentrations of HASMCs incubated with 100 nM Ang II and 200 nM thapsigargin for 16 h or only incubated with 100 nM Ang II for 16 h. n=6 per group. Student's t test, *$p < 0.05$.

I-J: Representative western blotting (I) and quantification (J) of HASMCs transfected with GSDMD vector, transfected GSDMD vector, and incubated with 200 µM TUDCA or transfected with empty vector for 48 h. n=8 per group. One-way ANOVA and Dunn post hoc test, *$p < 0.05$, ** $p < 0.01$.

K: LDH release of LPS/ATP-activated HASMCs transfected with GSDMD siRNA or NC siRNA after 12 h or 24 h. n=8 per group. Student's t test, ns: no significance.

L: The quantification of the ratio of p-C/EBPβ/ C/EBPβ. Student's t test.

M: The quantification of the ratio of p-NF-κB/ NF-κB. Student's t test.

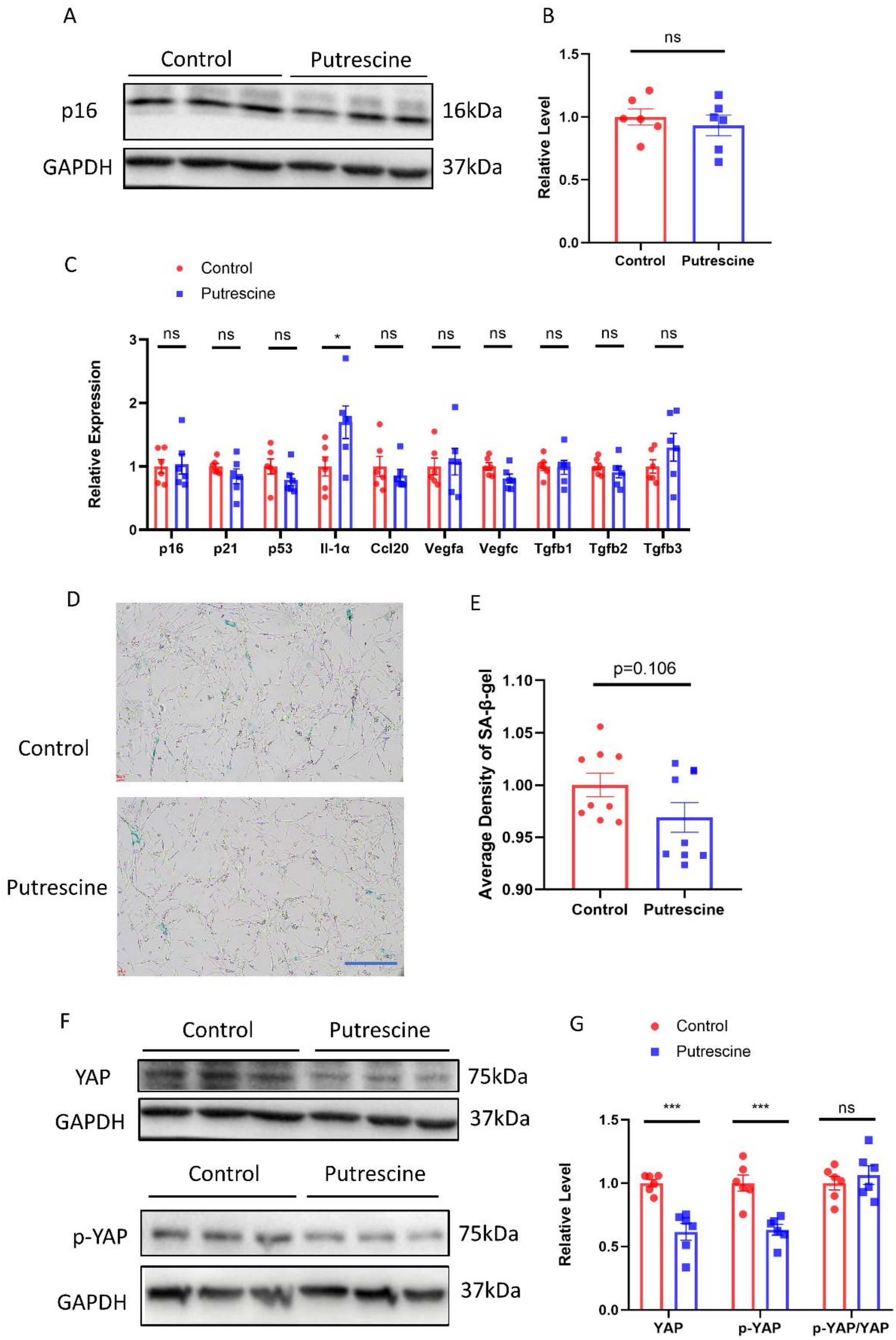

Supplemental Figure 5

A-B: Representative western blotting (A) and quantification (B) of p16 from HASMCs incubated with or without 50 μM putrescine for 24 h. n=6 per group. Student's t test, ns: no

significance.

C: mRNA abundance of a senescence-associated secretory phenotype related factors in HASMCs incubated with or without 50 μM putrescine for 24 h. n=5 per group. Student's t test, $*p < 0.05$, ns: no significance.

D: Senescence-associated beta galactosidase staining in HASMCs incubated with or without 50 μM putrescine for 24 h. n=9 per group. Student's t test.

F-G: Representative western blotting (F) and quantification (G) of YAP and p-YAP from HASMCs incubated with or without 50 μM putrescine for 24 h. n=6 per group. Student's t test, $***p < 0.001$, ns: no significance.
